# Regional and temporal heterogeneity of epithelial ovarian cancer tumor biopsies: implications for therapeutic strategies

**DOI:** 10.18632/oncotarget.10505

**Published:** 2016-07-09

**Authors:** Lara Paracchini, Laura Mannarino, Ilaria Craparotta, Chiara Romualdi, Robert Fruscio, Tommaso Grassi, Vittoria Fotia, Giulia Caratti, Patrizia Perego, Enrica Calura, Luca Clivio, Maurizio D’Incalci, Luca Beltrame, Sergio Marchini

**Affiliations:** ^1^Department of Oncology, IRCCS Istituto di Ricerche Farmacologiche “Mario Negri”, Milano, Italy; ^2^Department of Biology, University of Padova, Padova, Italy; ^3^Clinic of Obstetrics and Gynaecology, University of Milano-Bicocca, San Gerardo Hospital, Monza, Italy; ^4^PhD Program in Experimental Medicine, University of Pavia, Pavia, Italy; ^5^Pathology Unit, San Gerardo Hospital, University of Milan-Bicocca, Monza, Italy; ^*^Both are first authors; ^#^Both are last co-authors

**Keywords:** ovarian cancer, temporal heterogeneity, spatial heterogeneity, targeted next generation sequencing, drug resistance

## Abstract

Stage III/IV epithelial ovarian cancer (EOC) is a systemic disease. The clonal relationship among different tumor lesions at diagnosis (spatial heterogeneity) and how tumor clonal architecture evolves over time (temporal heterogeneity) have not yet been defined. Such knowledge would help to develop new target-based strategies, as biomarkers which can adjudge the success of therapeutic intervention should be independent of spatial and temporal heterogeneity.

The work described in this paper addresses spatial and temporal heterogeneity in a cohort of 71 tumor biopsies using targeted NGS technology. These samples were taken from twelve high grade serous (HGS) and seven non HSG-EOC, both at the time of primary surgery when the tumor was naïve to chemotherapy and after chemotherapy.

Matched tumor lesions growing in the ovary or at other anatomical sites show very different mutational landscapes with branched tumor evolution. Mutations in *ATM, ATR,* *TGFB3,* *VCAM1* and *COL3A1* genes were shared across all lesions. *BRCA1* and *BRCA2* genes were frequently mutated in synchronous lesions of non HGS-EOC. Relapsed disease seems to originate from resistant clones originally present at the time of primary surgery rather than from resistance acquired *de novo* during platinum based therapy.

Overall the work suggests that EOC continues to evolve. More detailed mapping of genetic lesions is necessary to improve therapeutic strategies.

## INTRODUCTION

The wealth of genomic data collected over the last 10 years has shed new light on the extent and clinical relevance of cellular heterogeneity in solid tumors. It is now well recognized that multiple genetically distinct sub-clones co-exist in the same clinical sample, a finding referred to as regional heterogeneity. Furthermore sub-clones often evolve by selective pressure during chemotherapy, following different evolutionary lineages, which constitutes temporal heterogeneity. Intra-tumor heterogeneity has been proposed as the main cause of treatment failure and drug resistance in many solid tumors [1].

The majority of patients with stage III-IV epithelial ovarian cancer (EOC), in particular high grade serous (HGS)-EOC, display disease in the ovary and extensive and multiple implantations sites disseminated in the abdominal cavity (synchronous lesions). Definition of the degree of tumor heterogeneity between biopsies taken from the ovarian mass and synchronous lesions and the evolution of sub-clonal populations emerging in the course of treatment would help to characterize the pattern of sensitivity/resistance to chemotherapeutic drugs. This knowledge might aid choosing the most appropriate therapy. The major impediments to acquiring such knowledge are the complex histopathological and molecular features of EOC. The term EOC is now considered misleading as it encompasses different diseases sharing the same anatomical site of growth but displaying distinct clinical behavior [2]. HGS-EOC is the most frequently diagnosed subtype. Low grade serous, endometrioid, mucinous and clear cell subtypes, with varying cellular grades, are less common and are collectively called “non HGS-EOC”.

Two studies have so far investigated regional heterogeneity in HGS-EOC using exome sequencing or whole genome SNP arrays [3, 4]. Both describe the divergence between primary and synchronous lesions in a subset of patients suggesting that the genomic profile of a single tumor biopsy taken from the ovary is not representative of the systemic nature of the disease. Little is known about the issue of temporal and regional heterogeneity in non HGS-EOC tumors. Our group has previously addressed, with targeted re-sequencing approach, temporal heterogeneity in both HGS and non HGS-EOC tumor biopsies, showing a low level of concordance in mutational profile between matched primary ovary and relapsed tumor biopsies [5].

The present study was performed to extend our understanding of effects of spatial and temporal heterogeneity on the sensitivity of HGS-EOC to platinum based therapy, and on resistance against it. The complete mutational profile of a panel of 65 genes of therapeutic and diagnostic interest was obtained with deep coverage sequencing. The study aims at determining the clonal relationship between primary malignancies in the ovary and matched synchronous lesions and inferring the evolutionary lineages between different primary lesions and matched relapsed disease after chemotherapy.

## RESULTS

### Cohort description and experimental design

**Figure 1:**
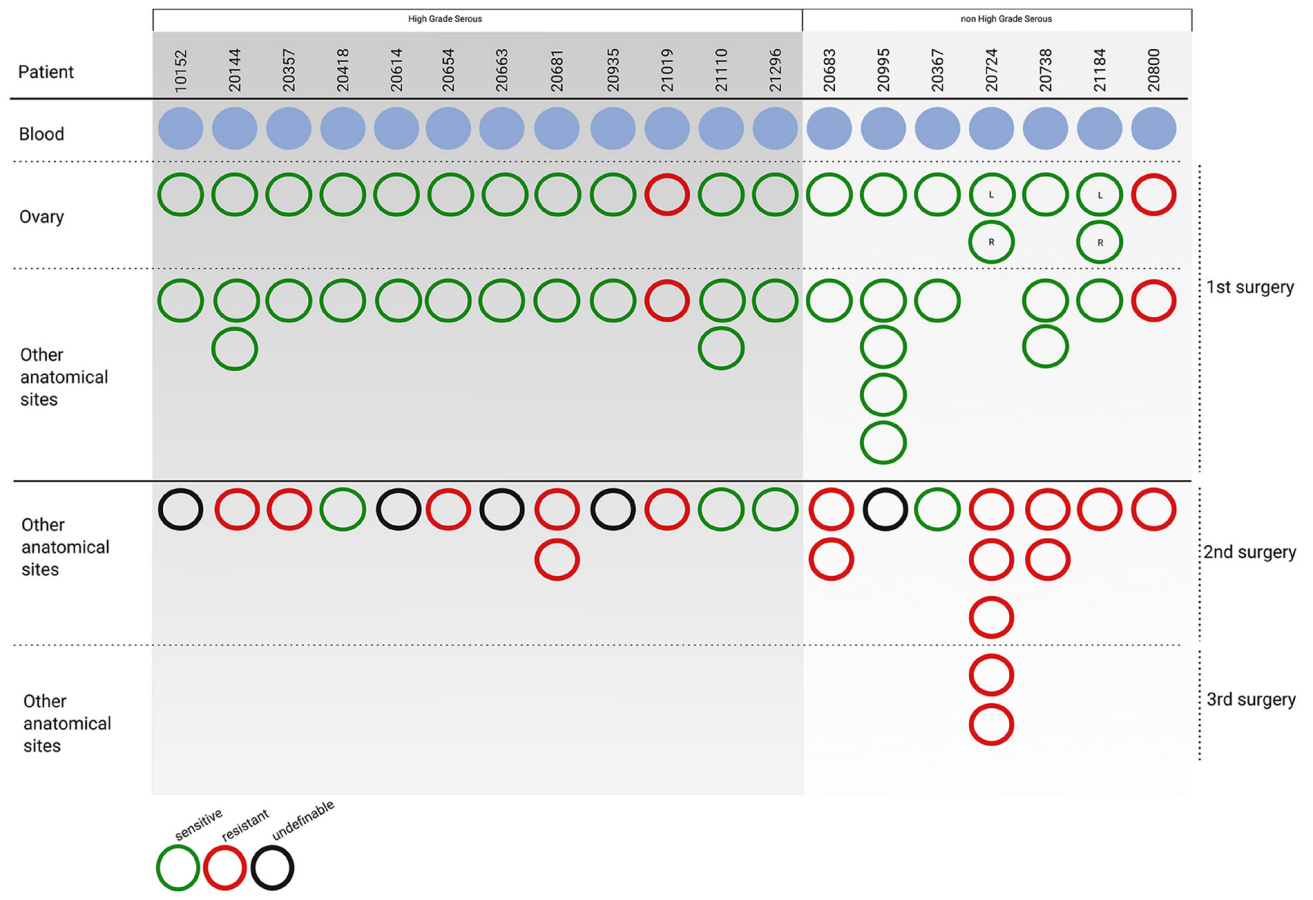
Patient cohort enrolled in the study. Graphical representation of clinico-pathological features of patients (*n* = 19) and tumor biopsies (*n* = 71) enrolled in the study. Matched blood samples (light blue circles, *n* = 19) were used as reference to exclude germline variants. At primary surgery, 21 samples were from the ovary, and 24 from different anatomical sites. After chemotherapy, 24 samples were from second surgery, while two samples from patient 20724, were at third surgery. Green circles, sensitivity to Pt-based treatment (PFS > 6 months from the end of chemotherapy); red circles, resistance against Pt-based treatment (PFS < 6 months from the end of chemotherapy). Black circles, information is missing. Tumors are grouped into high grade serous (*n* = 12) and non high grade serous (*n* = 7). Detailed anatomo-pathological features are reported in Supplementary Table 1.

To study regional and temporal heterogeneity, 71 samples from 19 EOC patients were analyzed across serous, endometrioid and mucinous tumors with different histological grades (Table 1). Cases were selected from the Pandora tumor tissue collection. Selection was based on three criteria, *i*) availability of whole blood samples at diagnosis; *ii*) availability of at least two different biopsies at primary surgery before patient treatment; *iii*) at least one matched biopsy at relapse, after one or more lines of chemotherapy. As summarized in Figure 1, 45 tumor biopsies were obtained at primary surgery (21 from the ovary and 24 from synchronous lesions), and 26 from follow-up surgery. The histological and clinical parameters are summarized in Table 1 and detailed in Supplementary Table 1. Briefly, cases selected for the study were all stage III/IV EOC, 63.2% were HGS-EOC (twelve out of 19), and 21.1% were low grade serous (LGS, four out of 19). Two high grade endometrioid cases (10.5%) and one mucinous case (5.2%) were included. The mean age of patients at diagnosis was 56 years. The mean follow-up was 4.5 years. In the case of patients 20724 and 21184 biopsies were taken from both left and right ovary at primary surgery. For patient 20724 follow up biopsies were obtained at both second and third surgery. As shown in Figure 1 and Table 1, at primary surgery two patients out of 19 (10.5%) were Pt-resistant, i.e., relapse occurred within 6 months from the end of Pt-based therapy. Seventeen patients (89.5%) were Pt-sensitive, i.e., relapse occurred beyond 6 months from the end of Pt-based therapy. At second surgery, ten patients (53%) were Pt-resistant, while four patients (21%) maintained Pt-sensitivity. Information is missing for five patients (26%, Figure 1).

**Table 1:** Patient characteristics

Clinical and pathological annotations	*N* of patients (%)
**Histotypes**	
Serous	16 (84.2)
Endometrioid	2 (10.5)
Mucinous	1 (5.3)
**Stage**	
III B	1 (5.3)
III C	14 (73.7)
IV	4(21.0)
**Grade**	
High	14 (73.7)
Low	5 (26.3)
**Anatomical sites analyzed**	
Ovary	21
Omentum	14
Peritoneum	2
Disseminated metastasis	8
**Pt- Sensitivity**	
Sensitive (PFS>6 months)	17 (89.5)
Resistant (PFS<6 months)	2 (10.5)
Mean age [min-max](years)	56 [28–79]
Mean follow-up [min-max](years)	4.5 [1–20]
Total number of patients	19

Histological and clinical annotation of patients enrolled in the study. Anatomical sites of biopsies analyzed and therapy response are referred to the first surgery. Abbreviation: Pt: platinum.

### Somatic variant call analysis

Analogous to our previously established pipeline of analysis [5], targeted re-sequencing technology was used to screen the coding sequences of 65 genes belonging to pharmacologically relevant pathways (Supplementary Table 3). For each patient, a matched blood sample was sequenced and used as reference to exclude germline variants from the analysis [5]. We identified a total number of 1131 somatic variants with wide inter- and intra-individual variation with a minimum coverage of 200 fold (200×) and an allelic fraction (AF) of >1% (Table 2 and Supplementary Section Methods 3.1.1). AF is the percentage of reads that carried the mutation in a single biopsy. The average number of somatic variant calls per patient in the ovary was 35.84 (±42.27), with an inter-individual range from 2 to 140. With regards to the synchronous lesions, the average was 18.00 (±9.44) with an inter-individual range from 0 to 37. The total number of shared mutations (defined as mutations present in at least one ovary and one synchronous disease at the same locus, although not necessarily in the same patient) was 108, representing 9.5% of the total number of identified variants. Somatic mutations were classified into four main groups: non synonymous mutations, synonymous mutations, insertions and deletions (indel, <50 bp) and variants of unknown significance (VUS). As shown in Table 2, VUS represented the highest number of mutations identified (*n* = 564, including shared mutations), of which only 13.3% were shared between ovaries and synchronous lesions. VUS identified exclusively in primary tumor site and in synchronous diseases were 337 and 152, respectively. As summarized in Supplementary Figure 1, for both HGS-EOC and non HGS-EOC subgroups, the percentages of VUS, non synonymous and synonymous mutations were comparable between lesions in the ovary and its matched synchronous disease. In both HGS-EOC and non HGS-EOC subgroups the number of indels was larger in synchronous lesions compared to primary tumors in the ovary (Supplementary Figure 1).

**Table 2:** Somatic mutation identified by the analysis

	Somatic mutations per patient	Inter-individual range	Non synonymous mutations	Synonymous mutations	Insertions deletions	VUS	Total
**Ovary**	35.84 ± 42.27	2–140	264	73	7	337	681
**Synchronous diseases**	18.00 ± 9.44	0–37	130	28	32	152	342
**Shared**	-	-	22	5	6	75	108
**Total**	-	-	416	106	45	564	1131

Mutations were classified as synonymous and non-synonymous, insertions and deletions (indels), and VUS (variant of unknown significance) which includes non-coding mutations mapping in intergenic regions, UTR or canonical splicing sites. Shared mutations are defined as mutation present at least in one ovary and in one synchronous disease in the same locus, but not necessary in the same patient. For ovary and synchronous lesions number of mutations for patients (± average) and inter-individual range are also reported.

In conclusion, analysis of the somatic variants revealed a marked level of intra-patient heterogeneity and among synchronous lesions from the same patient, i.e., regional heterogeneity. The latter finding warranted further analysis to study the effects of regional heterogeneity on therapeutic intervention.

### Regional heterogeneity

Stage III/IV EOC is a systemic disease characterized by multiple foci disseminated in the abdominal cavity. We initially reasoned whether the current lack of improvement in therapeutic strategies against EOC might be correlated with prevalence of sub-clones with different biological features in the ovary and matched synchronous lesions. To test this hypothesis, we consulted our somatic variants database with the aim of defining differences in mutational burden between primary tumor and synchronous lesions and assessing the level of similarity among multiple biopsies taken from the same patient.

**Figure 2:**
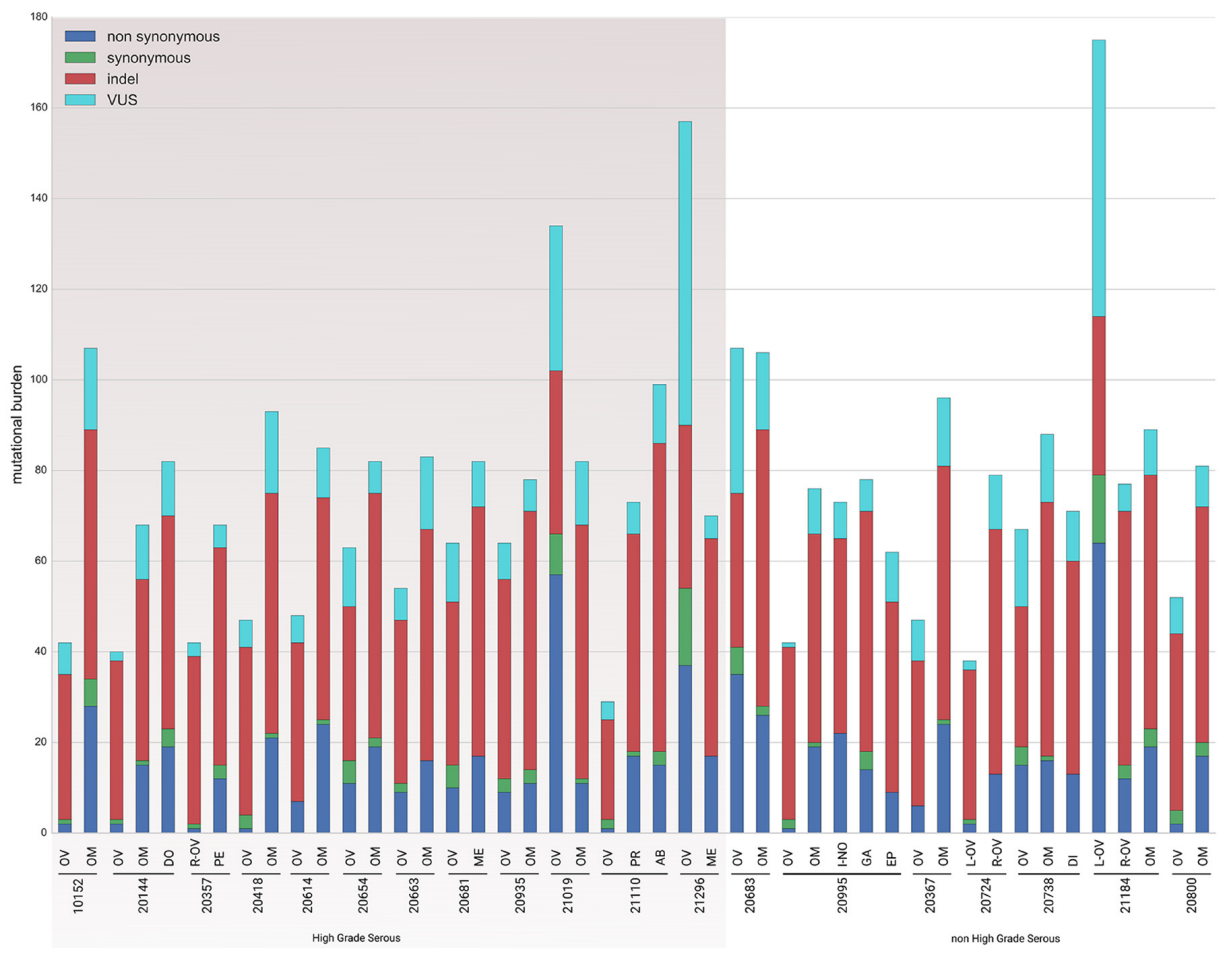
Mutational load. The diagram describes for each sample the mutational load. Mutations are categorized according to their predicted effect. Blue bars, non synonymous mutations; green bars, synonymous mutations; red bars, indel; light blue bars, VUS. Indel, insertion/deletion. VUS, variant of unknown significance. Complete list of sample names are reported in Supplementary Table 2.

As to the former aim, mutational burden is an indirect measure of evolutionary lineage in the tumor cells. It allows discrimination between the ovary and other anatomical sites in terms of tumor cell growth and replicative fitness, that is whether DNA damage occurred at the same time in different tumor lesions. For each patient, we plotted raw number of somatic mutations (i.e., non synonymous, synonymous, indel and VUS) in the ovary and in its matched synchronous lesions. Figure 2 shows, for both HGS-EOC and non HGS-EOC subgroups, a wide mutation burden per patient, regardless of anatomical site of growth. This result reflects the known genomic instability of stage III/IV EOC, because of defects in DNA repair paths are early during tumor growth. Indel and non synonymous mutations were the most abundant ones within each patient. Generally, the mutational burden observed in the ovaries was lower than that measured in the synchronous lesions. Results from the entire repertoire of somatic mutations suggest that within each patient evolutionary fitness of tumor cells differs between ovary and other anatomical sites. Thus, different tissue environments drive different evolutionary trajectories of tumor cells.

As to the second aim, we investigated the sub-clonal relationship between primary and synchronous lesions, through unsupervised cluster analysis on the AF of the 736 identified somatic variants across all samples. From this point onwards, synonymous mutations were excluded from the analysis. For both HGS-EOC (Figure 3A) and non HGS-EOC (Figure 3B), unsupervised cluster analysis depicted an unambiguous division of samples into branches I and III, including all synchronous lesions and branches II and IV consisting of ovarian tumor biopsies only. This finding suggests that at primary surgery, in both HGS and non HGS, pelvic–peritoneal implants are poorly correlated to their own matched ovarian lesions. Notably, for patients 20724 and 21184, both left and right ovaries were analyzed (Figure 1). Figures 2 and 3B show that tumor masses taken from the two gonads have a completely different mutational burden and mutational profile, suggesting that disease growth in the two different organs evolved differently. In the case of patient 20995 for whom multiple primary biopsies were available (Figure 1) synchronous lesions have comparable tumor loads with similar mutational profiles, and these are barely different from that in the ovary (Figures 2 and 3B).

**Figure 3A:**
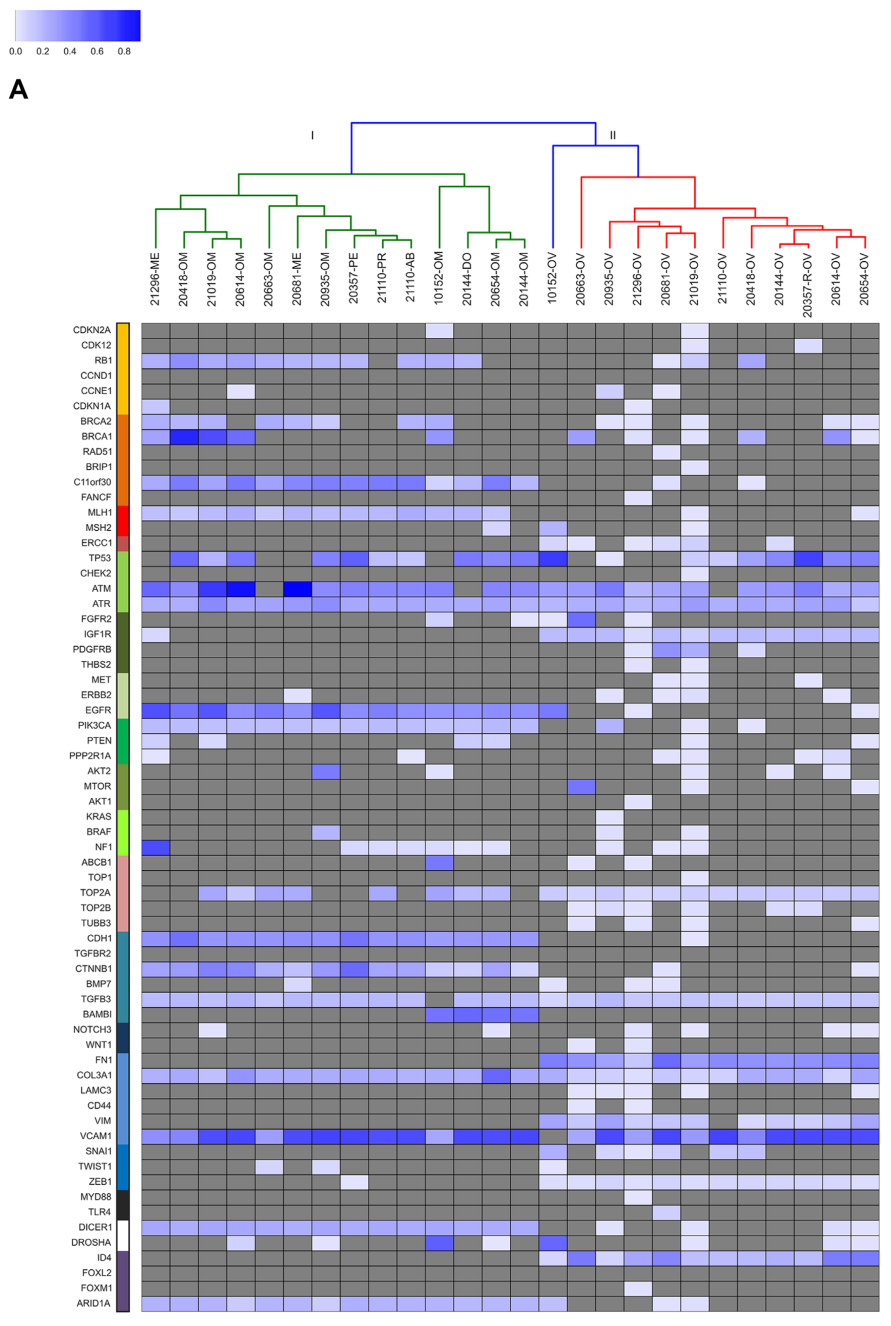
Unsupervised cluster analysis. Unsupervised clustering of somatic mutational allelic fractions (AF) depicted for HGS-EOC patients for each gene (row) and for each patient (column), AF is defined as the percentage of reads that carried the mutation versus the total reads. Complete list of sample names are reported in Supplementary Table 2.

**Figure 3B:**
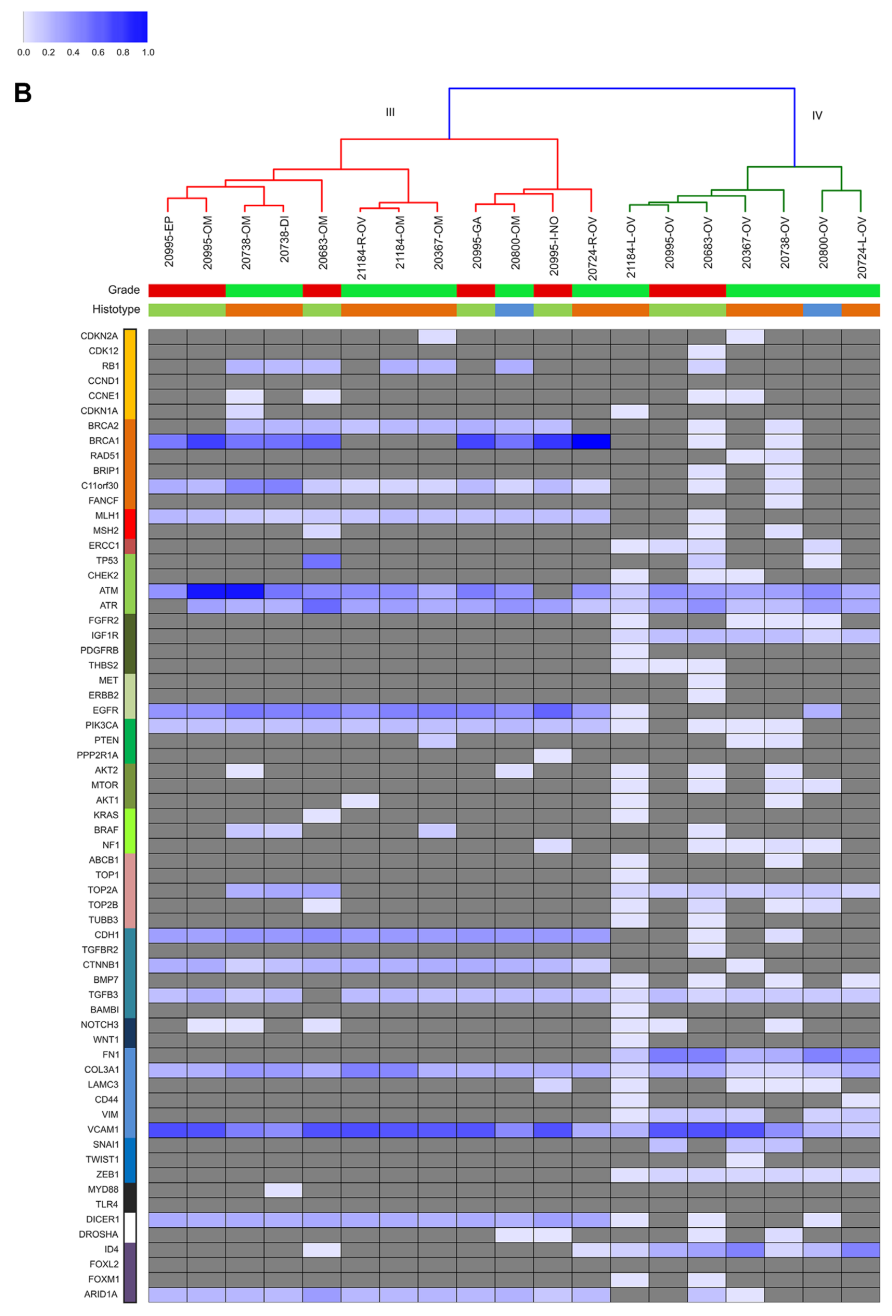
Unsupervised cluster analysis. Unsupervised clustering of somatic mutational allelic fractions (AF) depicted for non HGS-EOC patients. Color bars in the upper part of Panel B show information at diagnosis as reported in Table 1: grade (red, high grade; green, low grade) and histotype (orange, serous; green, endometrioid; blue, mucinous). For each gene (row) and for each patient (column), AF is defined as the percentage of reads that carried the mutation versus the total reads. Complete list of sample names are reported in Supplementary Table 2.

Further analysis (Figure 3A and 3B) shows that genes coding for *ATM, ATR, TGFB3, Col3A1,* and *VCAM1* were mutated nearly in all samples, independently of histo-pathological features and anatomical site of growth. Clusters I and III are characterized by mutations in genes such as *C11orf30, MLH1, EGFR, PIK3CA, CDH1, CTNNB1, DICER,* and *ARID1A*. Clusters II and IV, are otherwise characterized by mutations in genes such as *IGF1R, TOP2A, FN1, VIM, ZEB1* and *ID4*.

The aetiopathogenic role of *TP53* gene mutations and the therapeutic value of HR functional status are worthy to be analyzed in detail. Consistent with the literature, the *TP53* gene was mutated in the vast majority of ovarian HGS-EOC cases (cluster II). This was not observed in non HGS-EOC (IV). This data is commensurate with a role of TP53 in the early stages of tumorigenesis specific of HGS-EOC. It highlights that the non HGS-EOC are a different group of diseases at the molecular level. The frequency of *TP53* mutations in the HGS-EOC group was lower than that reported in the TCGA study (75% vs. 90%, respectively) [6]. This discrepancy may be due to the smaller sample size of our data set.

Considering the HR pathway, Figure 3 highlights two important results. Firstly, in HGS-EOC cases (Figure 3A) the mutational profile of the *BRCA1/2* genes in ovarian tumor biopsies barely mirrors that of their own synchronous lesions. Exceptions are patients 10152 and 20654. As to non HGS-EOC cases the *BRCA1/2* mutational status in the ovary does not reflect that of its own synchronous lesions. Thus, analysis of the ovarian tumor biopsy in non HGS-EOC would underestimate the fraction of patients eligible for therapeutic intervention. Secondly *C11orf30* known as *EMSY*, a *BRCA2* binding partner was found mutated in all synchronous lesions in both HGS and non HGS-EOC. This contrasts with primary ovarian tumor biopsies (wt status). These results are exemplified by analysis at pathway-based level, where all synchronous lesions of non HGS patients (cluster III) harbored a significantly greater number of mutations in the HR pathway, as opposed to their matched primary lesions (Supplementary Figure 2). These data suggest that analysis of genes belonging to HR pathway, rather than single *BRCA1* and BRCA1 evaluation, can be more informative to select those patients eligible for PARPi treatment.

The heatmap shown in Supplementary Figure 3 describes for each patient the distribution of mutations across tumor samples related to tumor grade and histology. Only two patients had *CCNE1* locus gene amplification (10152 and 20683) and one had *BRCA1* promoter hypermethylation. [7]. It has been described that loss of HR pathway is the major contribution to increased number of indels observed in EOC [8]. In our study, the increased number of indels counted in synchronous lesions compared to primary tumors (Supplementary Figure 1) can be justified by the frequent impairment of HR pathway observed in the synchronous lesions compared to primary tumors on the ovary (Supplementary Figure 2).

Regional heterogeneity between primary tumors and synchronous diseases was investigated at single gene level counting the number of mutations per gene. As shown in Supplementary Figure 4, ovaries and their synchronous diseases presented notable differences. In particular, for the majority of genes the biopsies taken from the ovaries were characterized by a higher number of mutations than the biopsies from synchronous lesions. *ATM*, *ATR* and *FN1* genes displayed a larger number of mutations in the lesions from the ovary than from other anatomical sites. Regional heterogeneity occurred also at pathway-based level (Supplementary Figure 2).

Collectively, these data reflect the unique evolutionary lineages of tumor cells growing in spatially different environments. This insight is inconsistent with the simplistic idea that targeting a single gene could affect tumor response of stage III/IV EOC.

### Concordant somatic mutations

To discriminate between “private” and “founder” mutations, we analyzed the rate of concordant mutations in tumor deposits in the ovary and in its pelvic-peritoneal environment. Concordant mutations are somatic variants in the same genetic locus in the ovary and at least in one of its matched synchronous lesions

**Figure 4:**
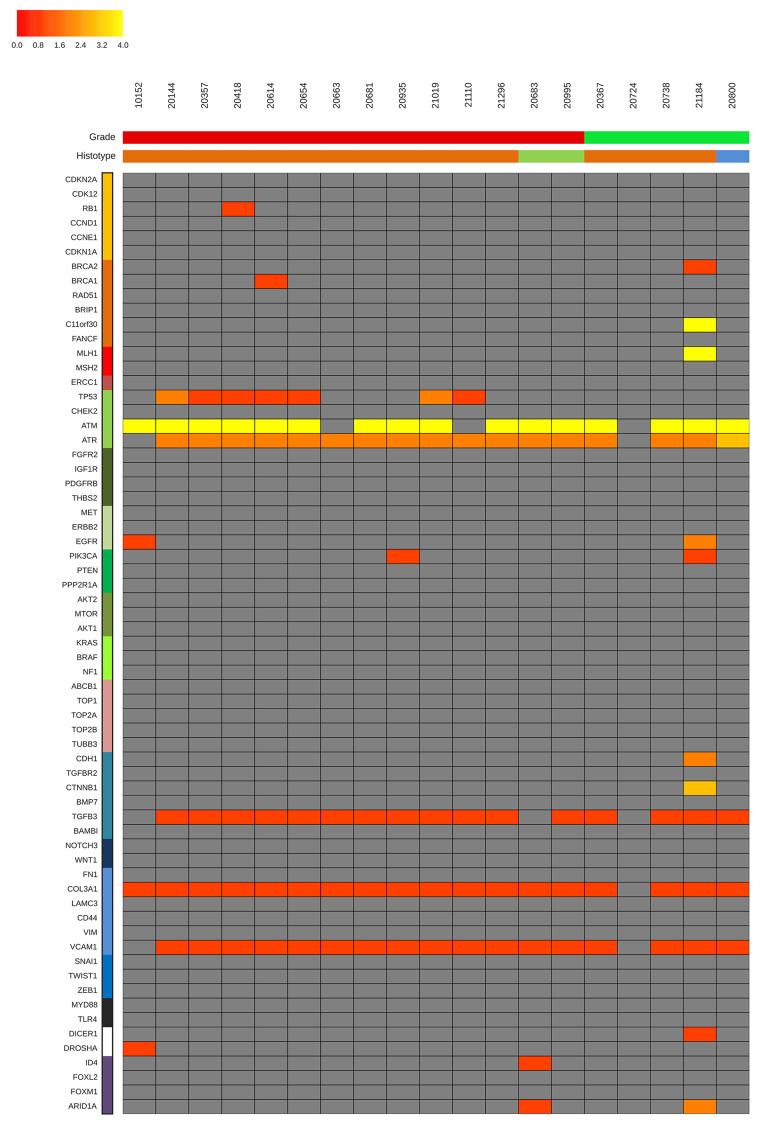
Concordant somatic mutations. Heatmap showing the distribution of concordant somatic mutations. The Number of concordant mutations summarized to single gene was reported for each patient in a false color scale. Grey boxes indicate the absence of concordant mutations. Genes are grouped into pathways, depicted by color palette, as described in Supplementary Table 2. Color bars in the upper part show information at diagnosis as reported in Supplementary Table 1: grade (red, high grade; green, low grade) and histotype (orange, serous; green, endometrioid; blue, mucinous).

The heatmap in Figure 4 shows the distribution of concordant somatic mutations (colored boxes) and the absence of concordant mutations (gray boxes) across matched tumor biopsies. The results demonstrate that each tumor deposit found at primary surgery is mainly composed of private mutations. Concordant mutations represent only 6.6% of all mutations passing filters. The complete list of concordant mutations, with their AF are shown in Supplementary Table 4. Pelvic-peritoneal implants are shown to harbor concordat mutations mainly in the genes, *TP53, ATM, ATR, TGFB3 Col3A1* and *VCAM1,* which suggests that populations of cancer cells obtained from different synchronous lesions are clonally correlated to an ancestral clone.

In conclusion, targeted re-sequencing analysis demonstrates marked intra-tumor heterogeneity. Although the primary ovarian tumor and synchronous lesions may harbor a huge amount of private genetic aberrations, the identification of founder genetic events define the sub-clonal relationship among ovarian cancer cells growing in the ovary or in other anatomical sites.

### Analysis of evolutionary lineages

**Figure 5:**
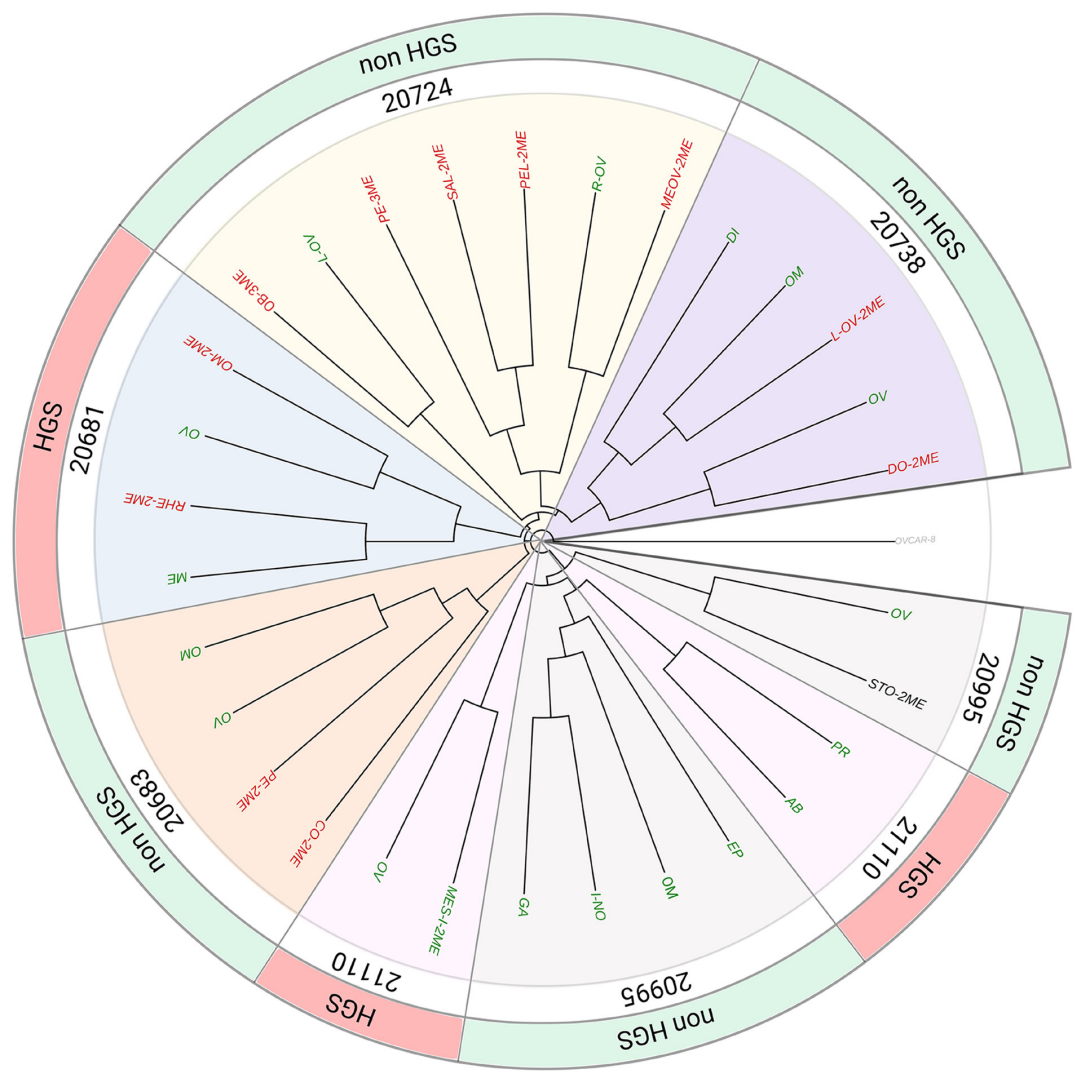
Phylogenetic tree. Phylogenetic tree depicting the clonal relationship among multiple biopsies at primary surgery (green leaves, originally pt sensitive) and at relapse (red leaves, platinum resistant or black, unknown) for both HGS and non-HGS EOC. The root of the tree is represented by OVCAR-8 cell lines used as unrelated control (see Supplementary Section Methods 3.1.2.4). The construction of the tree was based on mutant allelic fractions. Ovaries, synchronous diseases and metachronous diseases were considered. Complete list of sample names are reported in Supplementary Table 2.

Next, the ancestral clonal relationship was established between anatomical regions of primary and relapsed tumors reflecting temporal heterogeneity. To that end a phylogenetic tree was generated for each patient based on the allelic fractions on 4899 loci (Supplementary Methods 3.1.2.4). Figure 5 shows the evolutionary trees constructed for six patients (i.e., 20724, 20738, 20995, 21110, 20683, 20681) for whom samples from multiple sites were available. The complete list of AF data is available online (https://github.com/lbeltrame/mnegri-ov198). The phylogenetic trees for all samples are shown in Supplementary Figure 5.

Figure 5 depicts a branched evolutionary pattern, with multiple subclones evolving through different lineages. All synchronous lesions are derived from a common ancestral clone (tree trunk, Figure 5). The early branching among tumor samples indicates that the different tumor deposits diverge very early in their evolutionary histories, accompanied by the acquisition of a large number of private mutations during growth (Supplementary Table 5). This data are consistent with the previously reported marked intra-tumor heterogeneity of EOC [4, 5].

As to the relationship between relapsed-resistant tumor clones (red leaves, Figure 5) and matched primary sensitive deposits, relapsed clones appear in distant branches from the root. Resistant clones evolved probably from one disseminated clone in one of the primary tumor sites under selective pressure of chemotherapy, rather than *de novo*. Consistent with this interpretation platinum induced mutations were not detected in selected genes since the total number of single-base substitution (in particular C>T) were comparable before and after chemotherapy (Supplementary Figure 6).

We finally investigated the degree of similarity between samples in the leaves of the tree (Table 3). The results support the notion of early branching between samples, as the closest pairs in the tree had the majority of private mutations (> 90%) and only a limited number of concordant loci (between 0.17 and 3.59%). The same approach was used to compare ovaries with matched synchronous lesions or metachronous lesions, and to compare each pair of synchronous and metachronous lesions. Also here private mutations were prevalent (all above 90%) as compared to the low number of concordant loci (less than 1%) (Supplementary Tables 6–8).

**Table 3:** Similarity across the leaves of the phylogenetic tree

Leaf of the phylogenetic tree		Concordant mutations	Private mutations	Wt
**20995-OV**	**20995-STO-2ME**	0.21%	96.20%	3.59%
**21110-PR**	**21110-AB**	0.37%	95.45%	4.17%
**20995-I-NO**	**20995-GA**	0.39%	96.53%	3.07%
**21110-MES-I-2ME**	**21110-OV**	0.17%	97.36%	2.47%
**20683-CO-2ME**	**20683-OM**	0.44%	91.37%	8.20%
**20683-OV**	**20683-OM**	3.59%	96.41%	0.00%
**20681-ME**	**20681-RHE-2ME**	0.46%	97.72%	1.83%
**20681-OV**	**20681-OM-2ME**	0.35%	97.45%	2.20%
**20724-OB-3ME**	**20724-L-OV**	0.00%	98.96%	1.04%
**20724-SAL-2ME**	**20724-PEL-2ME**	1.00%	98.51%	0.50%
**20724-R-OV**	**20724-MEOV-2ME**	0.56%	97.55%	1.89%
**20738-OM**	**20738-L-OV-2ME**	0.48%	97.07%	2.45%
**20738-OV**	**20738-DO-2ME**	0.39%	97.97%	1.64%

Degree of similarity across the leaves of the phylogenetic tree including patients 20724, 20738, 21110, 20995, 20681 and 20683. *Leaf of the phylogenetic tree*, couple of analyzed samples; *Concordant mutations,* mutations present in both sample of the couple, in the same genomic locus; *Private mutations,* mutations present in only one sample of the couple. *Wt*, wild type. Complete list of sample names are reported in Supplementary Table 2.

## DISCUSSION

The results from this study reveal regional and temporal heterogeneity as a hallmark of both HGS and non HGS-EOC. There were extensive genomic differences, at both single nucleotide and pathway-based level, between tumor lesions growing in the ovaries and matched synchronous or metachronous lesions. In clinical practice, these findings have important implications. The primary lesion in the ovary is usually completely removed during debulking surgery, while adjuvant chemotherapy is largely targeted to eradicate microscopic or macroscopic residual tumor, which is one of the most important prognostic factor in stage III/IV EOC. Accurate and precise cytoreduction surgery aiming at reducing the number of cancer cells to “zero” is biologically important to lessen or delay the growth of the resistant clones that are still present in primary tumor lesions. The vast majority of patients relapse and die because of the re-growth of resistant disease. Genomic heterogeneity must be taken into consideration in order to improve the cure rate of EOC.

Until now, only few studies have addressed the degree of regional and temporal heterogeneity among different primary lesions of EOC, and how it could impact on the identification of reliable biomarkers for both diagnostic and prognostic purposes. Although this study analyzed a limited panel of genes across tumor biopsies taken from both HGS and non HGS-EOC, our results are consistent with previous studies in which small subsets of HGS-EOC were analyzed in terms of mutational landscape of the entire exome and global defects in genome architecture [3, 4, 9]. In the current study, a small subset of genes with therapeutic relevance for EOC were full length re-sequenced with deep coverage (mean depth of coverage of almost 2500×), to unmask those somatic variants present at very low AF within the tumor cell population as a source of temporal and spatial heterogeneity that could impact on therapeutic response.

The results suggest that biopsies taken at the time of primary surgery from the ovary and other anatomical sites developed along divergent evolutionary pathways. This notion suggests a “branching evolution” model, also often referred to as “parallel evolution” [10, 11]. This model suggests two important conclusions: 1) only few driver genes were found mutated in almost all samples, indicating that these are early events during tumor evolution. This is the case for example for the *TP53* gene in HGS-EOC patients, or in general, for the *ATM, ATR, TGF3 Col3A1* and *VCAM1* genes. 2) In both HGS and non-HGS-EOC the vast majority of individual variants have low AF, and tumor biopsies taken from ovaries and synchronous lesions cluster in two different branches. This result strongly suggests that both HGS and non HGS EOC evolve gradually by accumulating a large number of sub-clonal mutations. Each of them provides a relatively modest selection advantage, depending on different external and internal environmental factors. Relapsed disease arises probably not from new mutations but from resistant clones originally present in one of the primary lesions, the outgrowth of which is favored by the selective pressure of standard chemotherapeutic treatment.

These findings raise important issues with implications for the development of novel therapeutic strategies in stage III/IV EOC. Firstly, most of the translational studies performed so far in EOC largely ignored the bias of regional heterogeneity. It was thought that the molecular features of a tumor growing in the ovary largely mirrored the biology of the malignancy disseminated in the abdominal cavity. For this reason molecular features of synchronous disease have up to now been rarely studied. Our results show that synchronous lesions harbor a large amount of private somatic variants. This finding implies that dissecting the biology of the tumor in the ovary does not necessarily predict the therapeutic response of the different lesions spread to multiple sites within the abdominal cavity. The molecular information obtained in one biopsy does not necessarily reflect other tumor lesions. Therefore this information seems unlikely to help identify mutations in the ovary which can serve as biomarker predictors of response to molecular based target therapies.

The *ATM* gene seems worthy of discussion, as it is considered an attractive target for therapeutic intervention in EOC. In our study the *ATM* gene was mutated in almost samples from all patients, suggesting that loss of gene function is a founder event during tumor progression. The fact that we did not find variants in the relapsed tumor [5] intimates that clones harboring mutations in the *ATM* gene are sensitive to platinum based chemotherapy, irrespective of anatomical site. In the light of the recent emergence of targeted compounds that impair ATM protein function, it seems worthy of consideration to combine such ATM inhibitors with platinum-based therapy in both front line and subsequent chemotherapy.

Drug regulatory bodies such as FDA and EMA stipulate that identification of mutations in *BRCA1/2* genes should be exploited to select HGS-EOC patients for therapeutic intervention with novel PARP inhibitors [12]. Results from this study and others [5, 13] suggest the importance to move beyond the classical somatic and germline *BRCA1/2* analysis, to correlate the HR defects to response to treatment. For example, we have observed that all synchronous lesions enrolled in the study were characterized by genetic defects in at least one gene of the HR pathway, the one most frequently mutated being *C11orf30* (*EMSY*). These results suggest that the simple analysis of somatic *BRCA1/2* mutations in tumor biopsy taken from the ovary may well underestimate the fraction of patients who could benefit from treatment with PARPi drugs, irrespective of hystological subtype. As previously observed [5, 13] it may be prudent to include patients with either HGS and non HGS-EOC in future clinical trials with PARP inhibitors.

Finally the issue of temporal heterogeneity warrants discussion. We have previously shown that relapsed EOC disease shares less than 2% of concordant mutations with the primary tumor [5]. Therefore it remains unclear whether relapsed disease arises from clonal selection of pre-resistant clones in the original tumor mass or from accumulation of *de novo* mutations during platinum based treatment. Following the developmental route of the malignancy based on biopsies obtained from patients 20724 and 20738 at different times of treatment allows depiction of a phylogenetic tree. In this tree resistant tumors at relapse consist of clones selected for out of the many present in multiple deposits in the abdominal cavity. Consistent with the findings of Meier et al. [14] there was no evidence of enrichment of platinum drug-specific mutations in relapsed as compared to primary tumor. This result renders the possibility unlikely that resistant tumor clones arise from *de novo* mutations during therapy. As a corollary of this result, the use of targeted re-sequencing approaches at high depth of coverage is of utmost importance to uncover those mutations present at sub-clonal level in the primary tumor and with low AF that can expand during tumor re-growth after chemotherapy and characterize the genomic landscape of relapsed disease.

In conclusion, results from this study highlight three clinically important issues. *i*) sampling multiple sites at different time points can describes more accurately the genetic complexity of EOC tumor burden; *ii*) the genomic landscape of the ovarian tumor mass cannot be considered a suitable surrogate biomarker to aid prognosis in primary or relapsed disease; *iii*) attempts to develop novel therapeutic approaches in EOC should take intra-tumor heterogeneity among different tumor lesions into account. As EOC is a systemic disease, novel therapeutic strategies should be directed towards those genetic lesions that sustain tumor growth both in the ovary and other anatomical sites at the time of frontline therapy or relapse. There are some mutations in genes such as *C11orf30, MLH1, EGFR, PIK3CA, CDH1, CTNNB1, DICER,* and *ARID1A* which occur preferentially in synchronous lesions. Other genes such as *IGF1R, TOP2A,* *FN1, ID4, ZEB1* and *VIM* occur with higher abundance in the ovary. *iv*) Cytoreduction surgery which completely removes residual tumor is essential to reduce or delay selection of resistant clones that are probably still present in primary tumor lesions.

Analysis of molecular features of EOC using liquid biopsies taken at different times of follow-up might help to define more precisely the systemic nature of EOC and how it evolves over time.

## MATERIALS AND METHODS

### Patient cohort

A cohort of 19 stage III-IV EOC patients, from whom multiple snap frozen tumor biopsies (*n* = 71) were obtained at primary surgery from physically separated tumor sites and after one or more line of chemotherapy, were selected from the “Pandora” tumor tissue collection. The study was performed following the principles of the Declaration of Helsinki; the local scientific ethical committees approved the collection and usage of tumor samples. Written informed consent was obtained from all patients.

### Targeted resequencing libraries and massively parallel sequencing

gDNA was purified through an automatic nucleic acid purification system (Maxwell^®^ Rapid Sample Concentrator, Promega, Italy) as detailed in Supplementary Methods 1.1. Libraries for targeted re-sequencing of 65 selected genes, were generated using TrueSeq Custom Amplicon panel (TSCA, Illumina Palo Alto, CA, USA), with automatic liquid handling station (Epmotion 2075, Eppendorf, Italy), as previously described [5]. Quantified libraries were barcoded and sequenced on the MiSeq platform (Illumina) using the 2 × 150 bp configuration (23 × 23 cycles) and run on V2 sequencing flow cell. Details are reported in Supplementary Section Methods 2.1. The aligned sequences are available at the EBI European Nucleotide Archive (http://www.ebi.ac.uk/ena/data/view/; ID PRJEB6773 and PRJEB12935).

### Sequencing data analysis

Raw de-multiplexed reads from the MiSeq sequencer were aligned to the reference human genome (UCSC build hg19) using the Burrows-Wheeler Aligner (BWA, [15]). Putative somatic variant calls were detected with two separate programs, MuTect (version 1.1.5; [16]) and VarScan 2 (version 2.3.6; [17]), pairing each sample with its matched blood. Further details are available in Supplementary Methods 3.1.

## SUPPLEMENTARY MATERIALS











## Abbreviations

AF: allelic fraction
CR: complete response
EOC: epithelial ovarian cancer
FDR: false discovery rate
gDNA: genomic DNA
HGS-EOC: high grade serous ovarian cancer
HR: homologous recombination
LG-EOC: low grade epithelial ovarian cancer
NGS: next generation sequencing
PFS: progression free survival
PARP: poly(ADP-ribose) polymerase
PR: partial response
Pt: platinum
RECIST: response evaluation criteria in solid Tumors
VUS: variant of unknown significance
