# Supplementary material for: Regional and temporal heterogeneity of epithelial ovarian cancer tumor biopsies: implications for therapeutic strategies
Source: Oncotarget. 2016 Jul 9;12(24):2404–17. doi: 10.18632/oncotarget.10505 (PMC8629406; doi:10.18632/oncotarget.10505)
Supplement: Supplementary file 1 [file oncotarget-12-2404-s001.pdf]

Regional and temporal heterogeneity of epithelial ovarian cancer tumor biopsies: implications for therapeutic strategies

SUPPLEMENTARY MATERIALS

SUPPLEMENTARY RESULTS

Somatic variant call analysis

Considering HGS-EOC (Panel A and B) and non HGS-EOC patients (Panel C and D), somatic variants identified in the ovary (primary tumor site) and in matched synchronous lesions were functionally subdivided into: non synonymous coding mutations, synonymous coding mutations, insertion and deletions (indels) and variants of unknown significance (VUS). Pie charts in Supplementary Figure 1 show the percentage for each type, counted in both the ovary and in its own synchronous lesions. Variants in the *1000 Genomes Project* were filtered out from the analysis.

Regional heterogeneity at the pathway level

We next shifted our attention from genes relevant to therapeutic strategies towards the pathways in which mutated genes are involved. Data reported in in Figure 3 were collapsed into the 19 pathways previously described (Supplementary Method 3.1.2.7). Supplementary Figure 2 reports in a false color scale the prevalence of mutated pathways according to clusters I and II, III and IV previously obtained (Figure 3A and Figure 3B, respectively). In both HGS and non HGS group, there are differences between tumor lesions growing in the ovary or in other anatomical sites. Synchronous lesions were mainly characterized by mutations in homologous recombination, mismatch repair and miRNA biogenesis pathways. Differently, tumor biopsies withdrawn in the ovary are characterized by defects in signal transduction, drug resistance and EMT-TF pathways. Student's *t*-test analysis confirmed that these pathways were significantly different between synchronous lesions and ovaries, in both HGS and non HGS group (FDR < 0.01). On the other hand, DNA damaging signaling, TGF pathway, ECM interaction and transcription regulation were found ubiquitously mutated.

Allelic fraction heatmap

The allelic fractions (AFs) of the 736 identified somatic variants are shown in a false color scale heatmap. For each patient, tumor samples taken from different anatomical sites at primary surgery are grouped together accordingly to their histological features (histotype and grade). CCNE1 locus gene amplification and methylation status of BRCA1 promoter have been reported in literature [1] as poor prognostic markers in HGS-EOC. In our cohort of patients, with the exception of patient 10152, regional heterogeneity does not affect the locus gene amplification for *CCNE1* or the promoter methylation status of *BRCA1* gene.

Mutational load

For each gene, the total number of somatic mutations identified in ovary and in their matched synchronous lesions were counted. Data reported in Supplementary Figure 4 shows that synchronous lesions are characterized by increased number of somatic mutations compared to their matched primary tumor site in the ovary. The *ATM*, *ATR* and *FN1* genes resulted more frequently mutated.

Phylogenetic trees

In order to decipher temporal and spatial heterogeneity, a phylogenetic tree was depicted for each patient based on the allelic fraction of identified somatic mutations, as described in Supplementary Methods 3.1.2.4.

Analysis of mutational signatures related to chemotherapy exposure

To asses whether at relapse somatic mutations were acquired *de novo* or outgrowth by sub-clonal selections, we focused our attention on the total number of C>T, or G>A transition counted at primary surgery or at relapse. It has been reported that Pt-treatment is able to induce single base substitutions [2]. In Supplementary Figure 6 we compared the number of base changes in biopsies taken at first surgery (both ovary and synchronous lesions) with those taken at second/third surgeries (metachronous diseases) after Pt-based therapies. Results show no differences between the two groups suggesting that the high level of private mutations counted in the synchronous lesions are due to selection of clones originally present in the tumor cell populations rather than *de novo* acquisition.

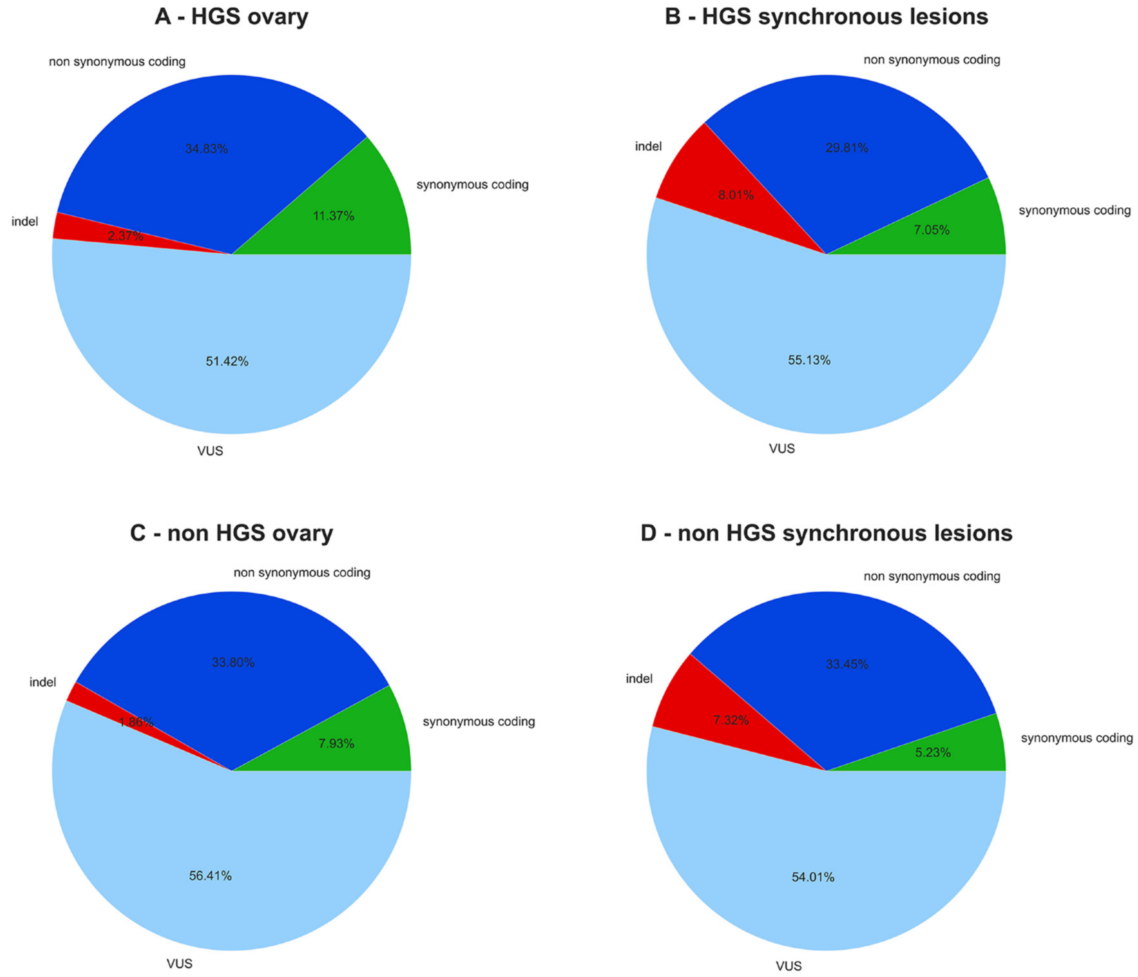

Supplementary Figure 1: Functional characterization of somatic variants called in the ovary (**A** and **C**) and in synchronous diseases (**B** and **D**), for both HGS-EOC (upper panel) and non HGS-EOC (lower panel) patients. Indel, insertion/deletion, VUS, variants of unknown significance.

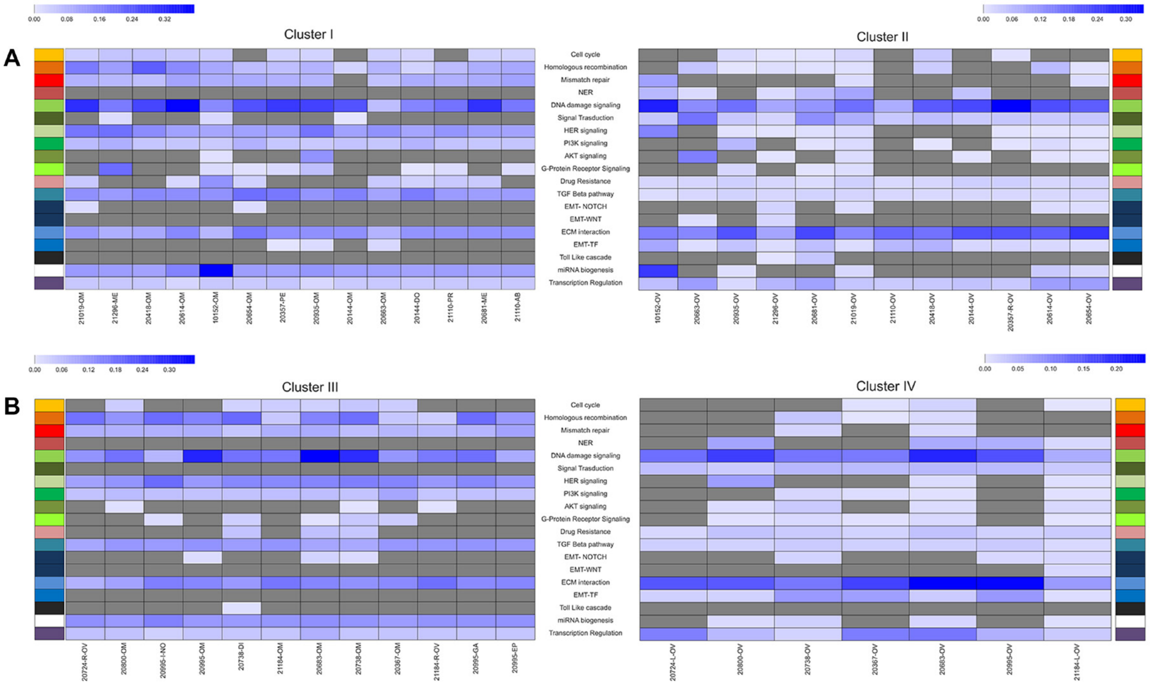

Supplementary Figure 2: Pathways mutational state. The mutational state of each pathway is based on the allelic fraction and computed as reported in Supplementary Section Methods 3.1.2.7. Fractions are reported for each patient in a false color scale. (**A**) Pathways analysis for HGS-EOC samples, clustered into cluster I or cluster II, as reported in Figure 3A. (**B**) Pathways analysis for non HGS-EOC samples clustered into cluster III and cluster IV as reported in Figure 3B. Grey boxes indicate wild type sequence. Pathways are depicted by color palette, as described in Supplementary Table 3.

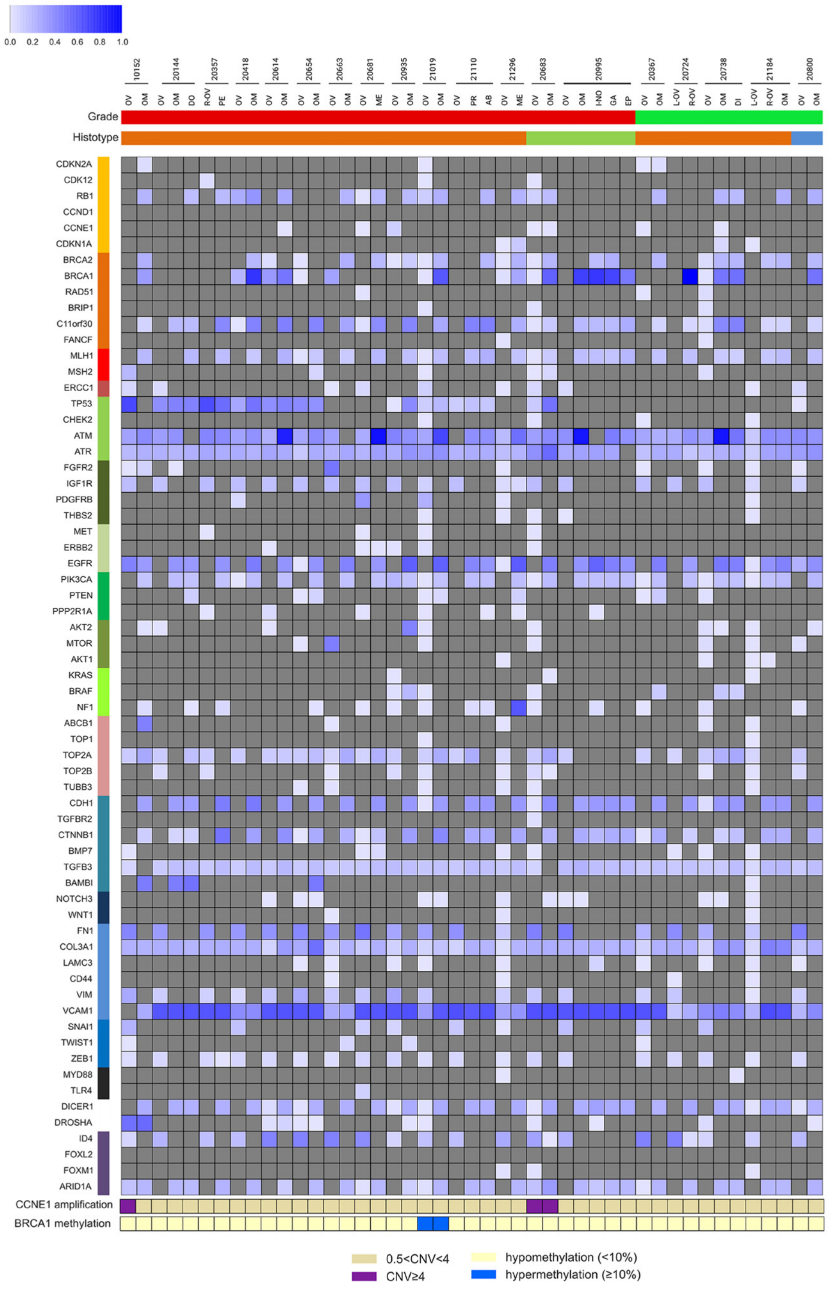

Supplementary Figure 3: Heatmap of the 736 somatic variants based on their allelic fraction. Fractions are reported for each patient in a false color scale; grey boxes indicate absence of variants. Genes are grouped into pathways, depicted by color palette, as describe in Supplementary Table 3. Color bars in the upper part show histopatological information at diagnosis as reported in Supplementary Table 1 (and as previously reported [1]). In particular, grade (red, high grade; green, low grade) and histotype (orange, serous; green, endometrioid; blue, mucinous).

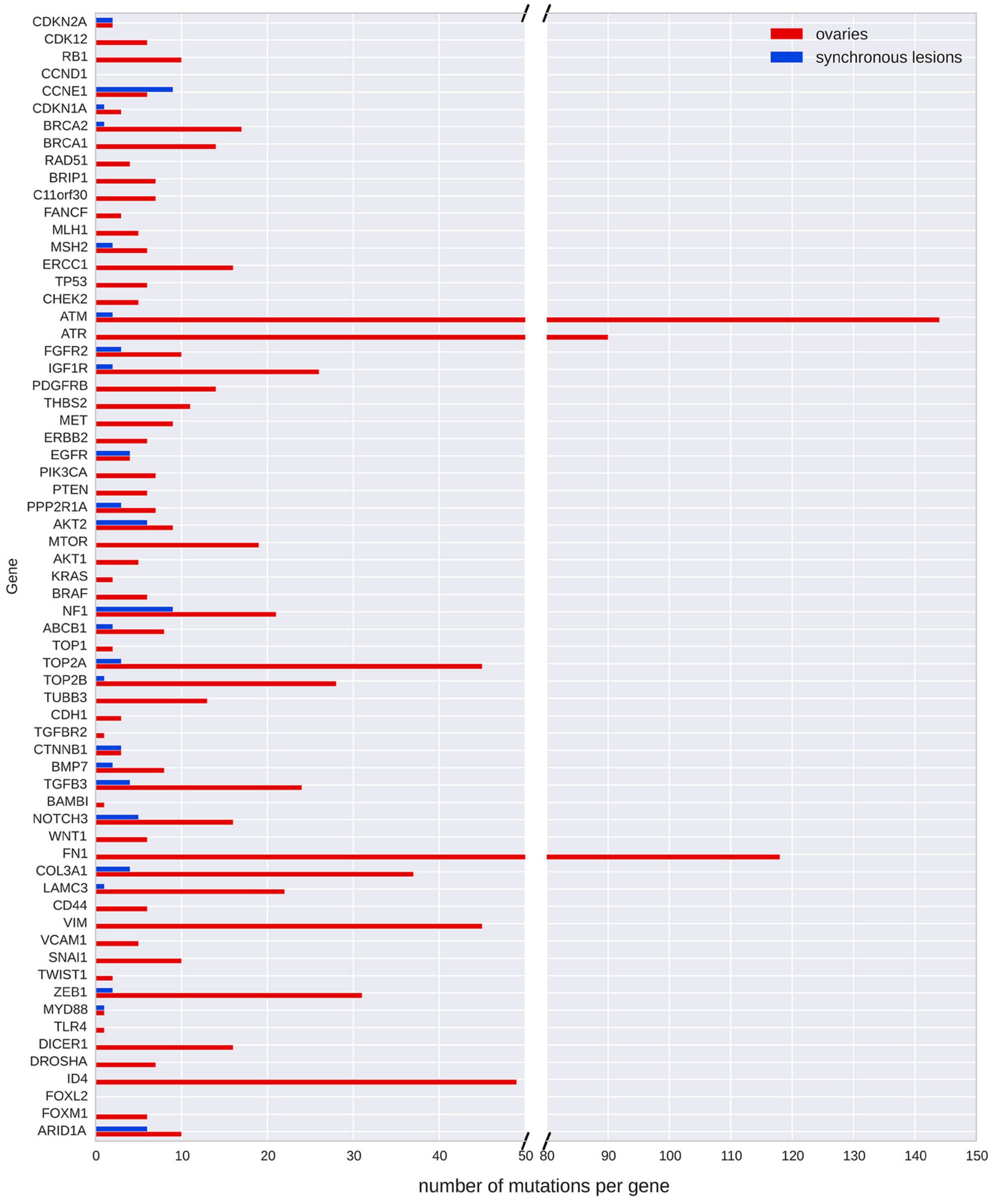

Supplementary Figure 4: Box plot summarizing for each gene the total number of somatic mutations counted in the ovary (red) and in synchronous lesion (blue). Shared mutations are not counted. Genes are grouped into pathways, depicted by color palette, as describe in Supplementary Table 3.

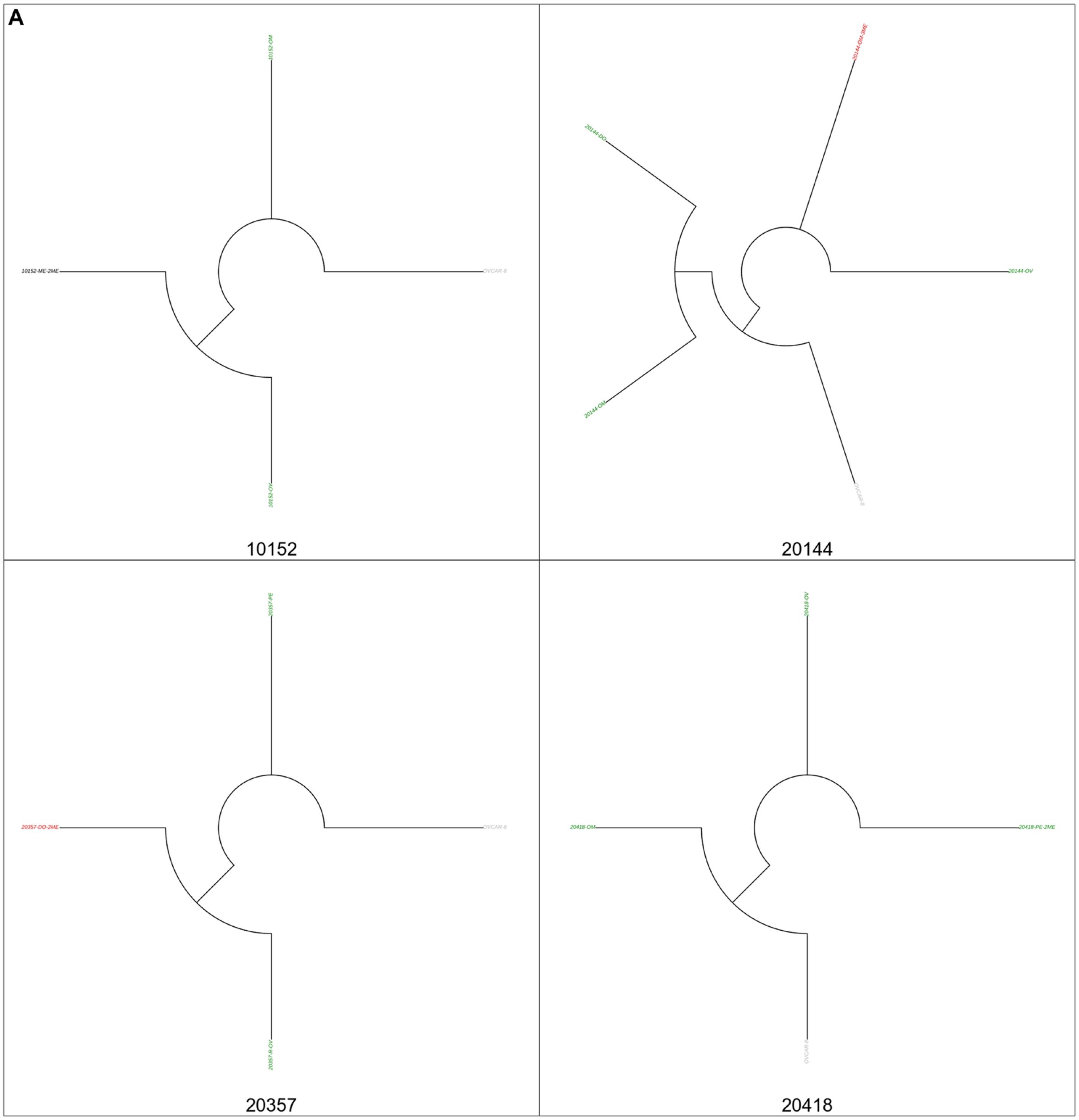

Supplementary Figure 5: (**A**) Phylogenetic trees for each high grade serous (HGS) patient computed as described in Supplementary Section Methods 3.1.2.4. Green leaves, sensitivity to Pt-based treatment (PFS > 6 months from the end of chemotherapy); red leaves, resistance to Pt-based treatment (PFS < 6 months from the end of chemotherapy). Black, no information available about Pt-based therapy response. (*Continued*)

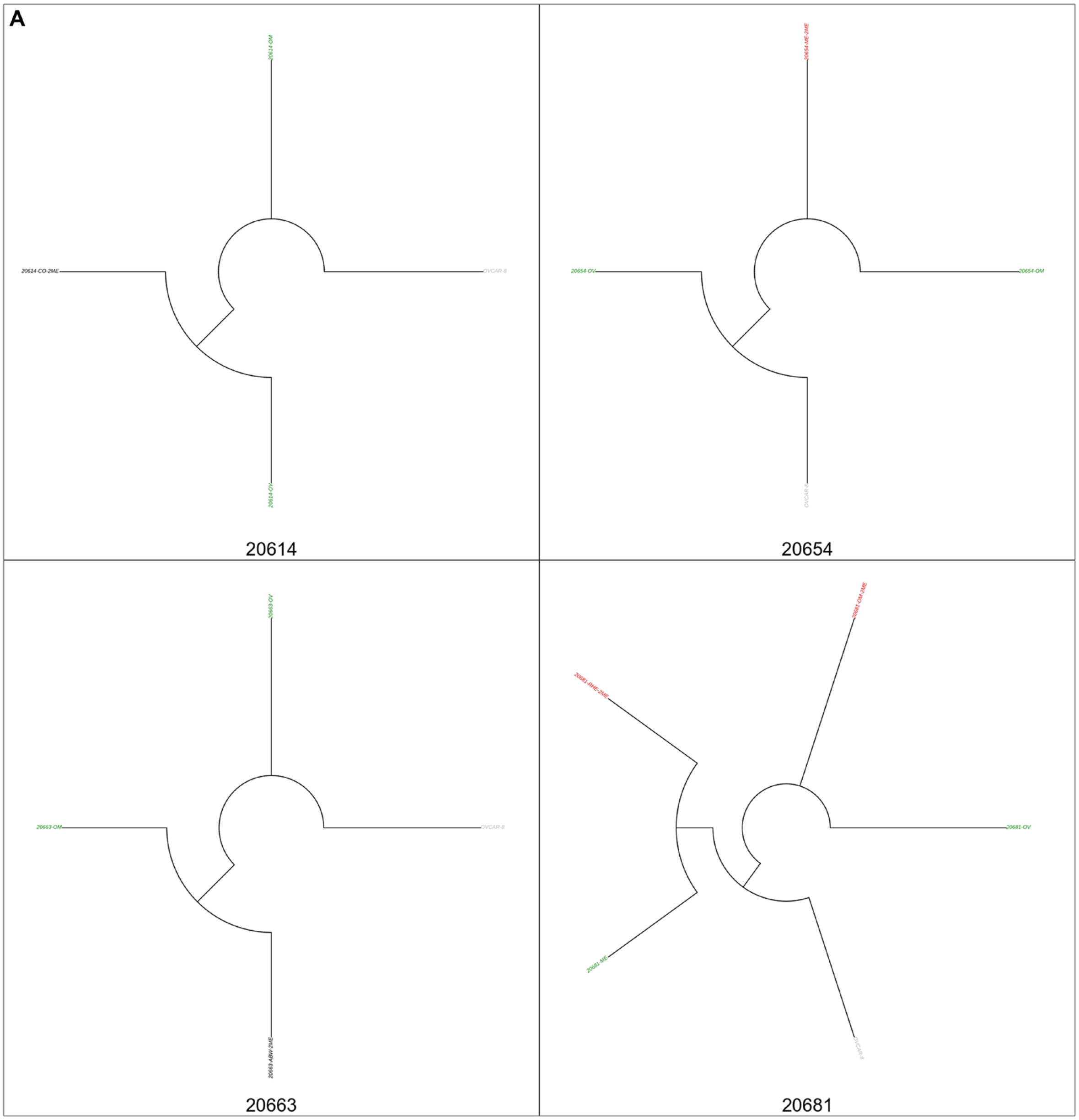

Supplementary Figure 5: (**A**) Phylogenetic trees for each high grade serous (HGS) patient computed as described in Supplementary Section Methods 3.1.2.4. Green leaves, sensitivity to Pt-based treatment (PFS > 6 months from the end of chemotherapy); red leaves, resistance to Pt-based treatment (PFS < 6 months from the end of chemotherapy). Black, no information available about Pt-based therapy response. (Continued)

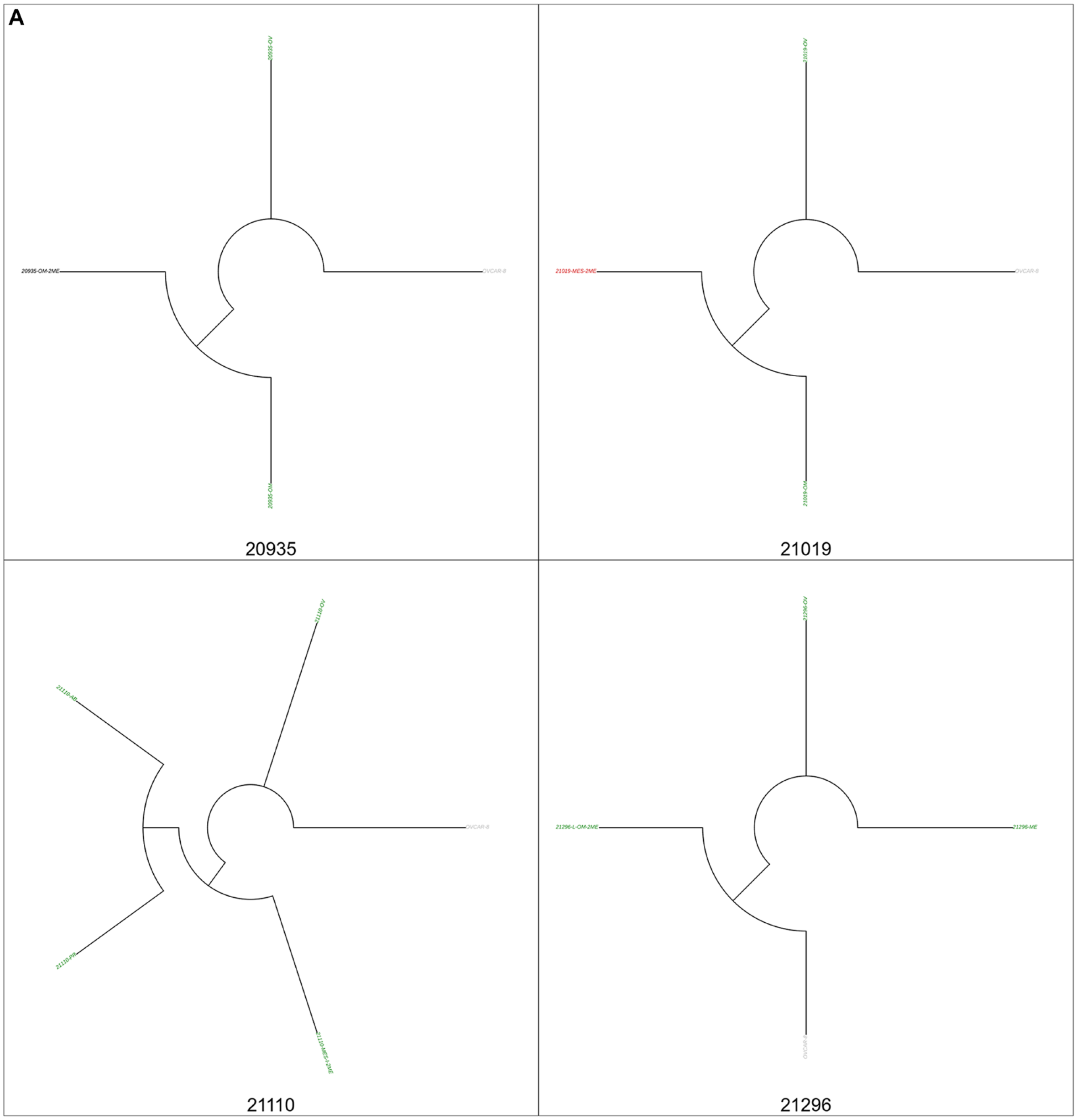

Supplementary Figure 5: (**A**) Phylogenetic trees for each high grade serous (HGS) patient computed as described in Supplementary Section Methods 3.1.2.4. Green leaves, sensitivity to Pt-based treatment (PFS > 6 months from the end of chemotherapy); red leaves, resistance to Pt-based treatment (PFS < 6 months from the end of chemotherapy). Black, no information available about Pt-based therapy response.

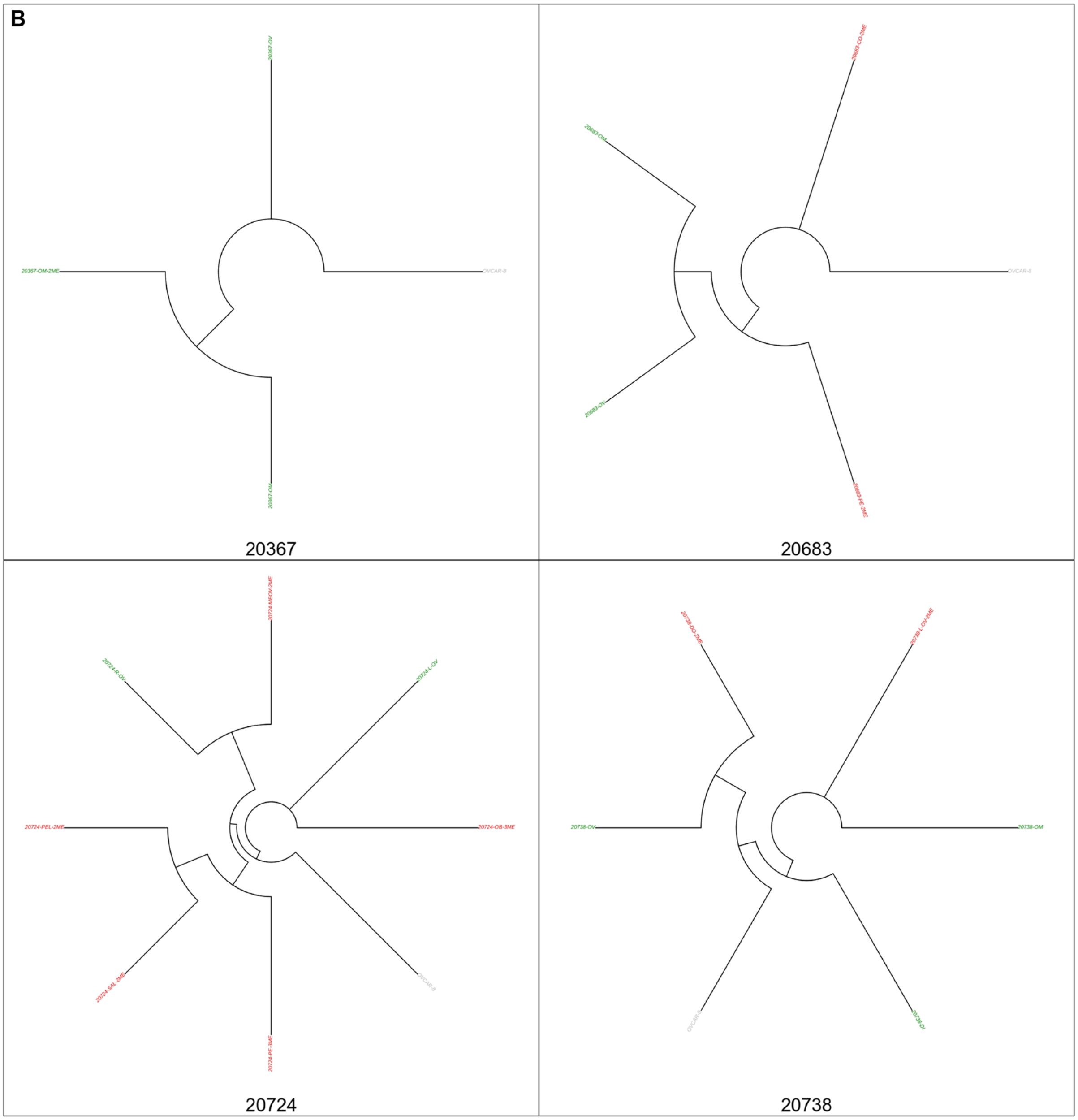

Supplementary Figure 5: (**B**) Phylogenetic trees for each non high grade serous (non HGS) patient computed as described in Supplementary Section Methods 3.1.2.4. Green leaves, sensitivity to Pt-based treatment (PFS > 6 months from the end of chemotherapy); red leaves, resistance to Pt-based treatment (PFS < 6 months from the end of chemotherapy). Black, no information available about Pt-based therapy response. (*Continued*)

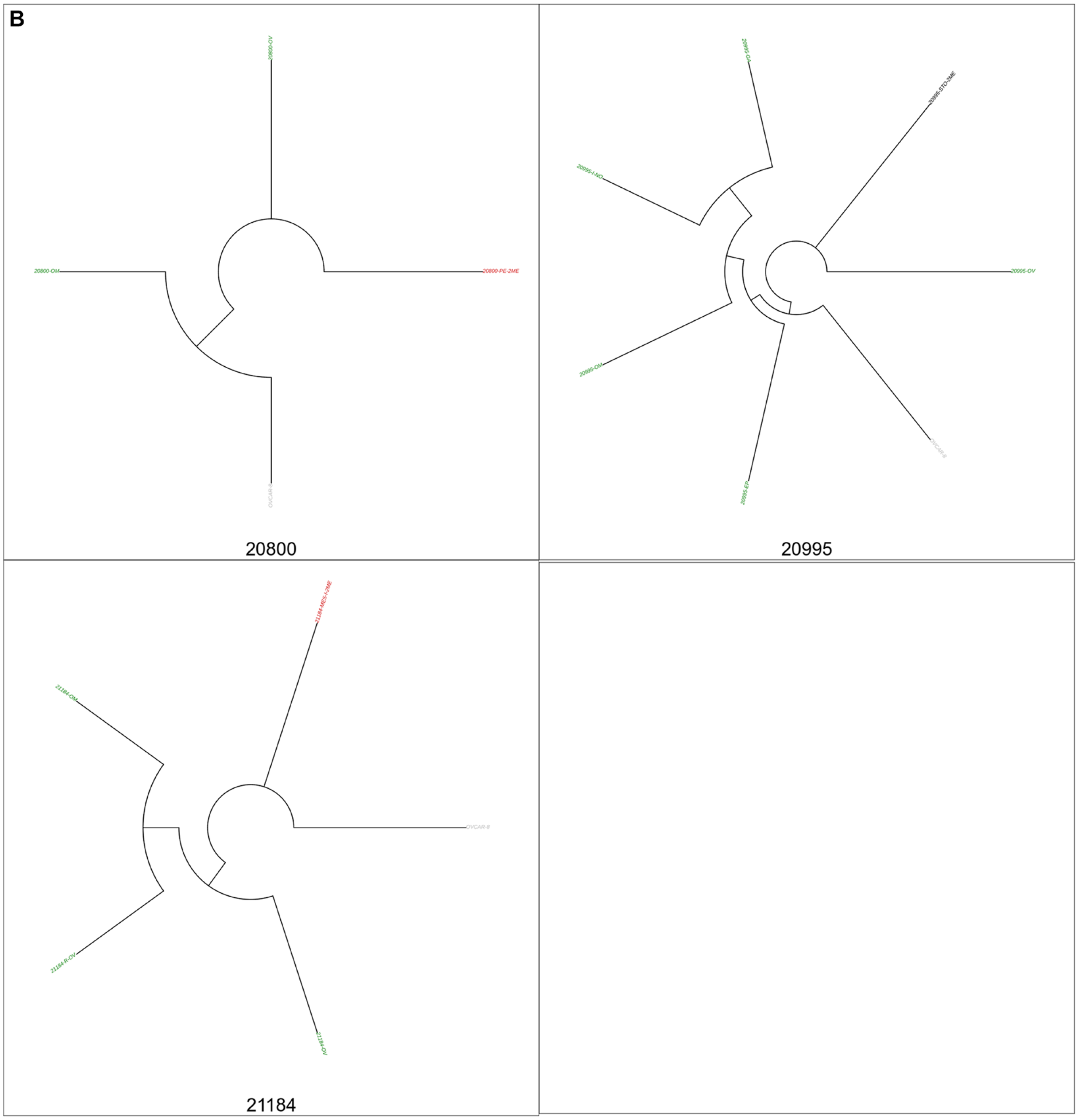

Supplementary Figure 5: (**B**) Phylogenetic trees for each non high grade serous (non HGS) patient computed as described in Supplementary Section Methods 3.1.2.4. Green, sensitivity to Pt-based treatment (PFS > 6 months from the end of chemotherapy); red, resistance to Pt-based treatment (PFS < 6 months from the end of chemotherapy). Black, no information available about Pt-based therapy response.

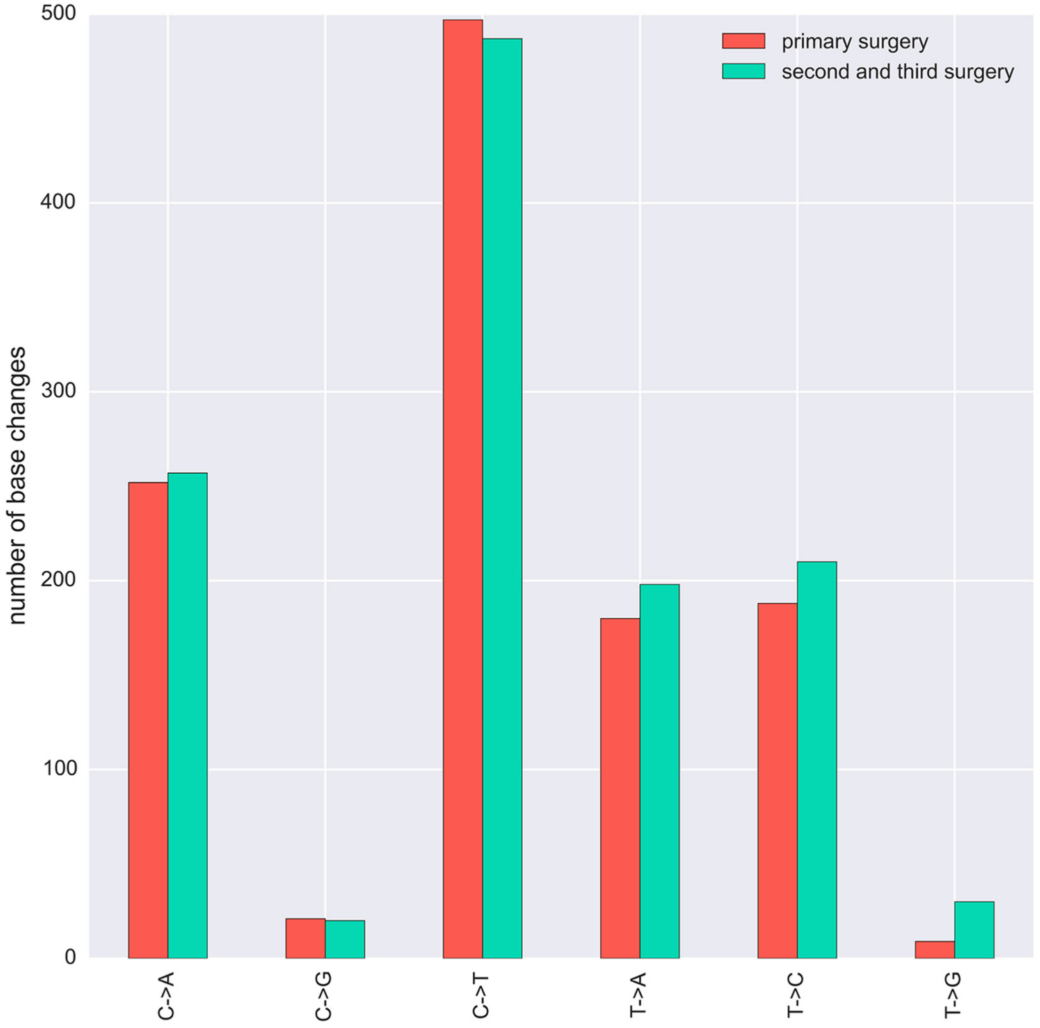

Supplementary Figure 6: Quantification of base changes primary surgery (ovaries plus synchronous lesions) (red) and at relapse (green).

Supplementary Table 1: Detailed description of patients enrolled in the study. Detailed clinical and pathological description of the cohort of patients enrolled in the study. CBDCA, carboplatin. Tax, taxol. Gem, gemcitabine. CDDP, cisplatin. PAC, paclitaxel. TPT, topotecan. TEP, paclitaxel, epirubicin and cisplatin. HD, high dose. Doxil, pegylated liposomal doxorubicin. According to RECIST criteria, clinical response was classified as complete (cCR) or partial response (pPR). NED, no evidence of disease. Residual tumor, is the tumor mass left in the abdomen after cytoreduction surgery. For each patient, both primary and second or third surgery sites are indicated. See Supplementary Table 1

Supplementary Table 2: Anatomical sites, list of abbreviations.

Patient Code	Histological Site	Abbreviation used	Surgery	N Samples
**10152**	metachr	10152-ME-2ME	2	3
	omentum	10152-OM	1	
	ovary	10152-OV	1	
**20144**	douglas	20144-DO	1	4
	omentum	20144-OM-2ME	2	
	omentum	20144-OM	1	
	ovary	20144-OV	1	
**20357**	douglas	20357-DO-2ME	2	3
	peritoneum	20357-PE	1	
	right	20357-R-OV	1	
**20367**	omentum	20367-OM-2ME	2	3
	omentum	20367-OM	1	
	ovary	20367-OV	1	
**20418**	omentum	20418-OM	1	3
	ovary	20418-OV	1	
	peritoneum	20418-PE-2ME	2	
**20614**	colon	20614-CO-2ME	2	3
	omentum	20614-OM	1	
	ovary	20614-OV	1	
**20654**	metachr	20654-ME-2ME	2	3
	omentum	20654-OM	1	
	ovary	20654-OV	1	
**20663**	abdominal	20663-ABW-2ME	2	3
	omentum	20663-OM	1	
	ovary	20663-OV	1	
**20681**	ovary	20681-OV	1	4
	right	20681-RHE-2ME	2	
	synchr	20681-ME	1	
	omentum	20681-OM-2ME	2	
**20683**	colon	20683-CO-2ME	2	4
	omentum	20683-OM	1	
	ovary	20683-OV	1	
	peritoneum	20683-PE-2ME	2	
**20724**	left	20724-L-OV	1	7
	mesovarium	20724-MEOV-2ME	2	
	pelvis	20724-PEL-2ME	2	

Patient Code	Histological Site	Abbreviation used	Surgery	N Samples
	peritoneum	20724-PE-3ME	3	
	right	20724-R-OV	1	
	salpinx	20724-SAL-2ME	2	
	obturator	20724-OB-3ME	3	
**20738**	diafram	20738-DI	1	5
	left	20738-L-OV-2ME	2	
	omentum	20738-OM	1	
	ovary	20738-OV	1	
	douglas	20738-DO-2ME	2	
**20800**	omentum	20800-OM	1	3
	ovary	20800-OV	1	
	peritoneum	20800-PE-2ME	2	
**20935**	omentum	20935-OM-2ME	2	3
	omentum	20935-OM	1	
	ovary	20935-OV	1	
**20995**	epiploic	20995-EP	1	6
	gastric	20995-GA	1	
	ileum	20995-I-NO	1	
	omentum	20995-OM	1	
	ovary	20995-OV	1	
	stomach	20995-STO-2ME	2	
**21019**	mesocolon	21019-MES-2ME	2	3
	omentum	21019-OM	1	
	ovary	21019-OV	1	
**21110**	abdominal	21110-AB	1	4
	mesoileum	21110-MES-I-2ME	2	
	ovary	21110-OV	1	
	prevesical	21110-PR	1	
**21184**	mesoileum	21184-MES-I-2ME	2	4
	omentum	21184-OM	1	
	ovary	21184-OV	1	
	right	21184-R-OV	1	
**21296**	little	21296-L-OM-2ME	2	3
	ovary	21296-OV	1	
	synchr	21296-ME	1	

Table reports for each patient enrolled in the study, the complete list abbreviations used for the different anatomical sites investigated by NGS technology.

Supplementary Table 3: Genes and pathways. The complete list of 65 genes selected for the study has been organized into functional pathways and the list of known drugs targeting the gene are reported. Color palette column is used through the text for the graphical representation of pathways under analysis. Association with drugs was evaluated with QIAGEN's Ingenuity® Pathway Analysis (IPA®, QIAGEN Redwood City, http://www.qiagen.com/ingenuity). See Supplementary Table 3

Supplementary Table 4: List of concordant variants. For each variant is reported: chromosome (chrom.), the 0-based start position (start), and the 1-based end position (end); gene name (gene), reference allele (ref); alternate allele (alt); state of the filter (pass or not); variant type and subtype; the consequence of the affected transcript (impact); the codon change; the amino acid change (aa_change); the polymorphism phenotyping prediction (polyphen_prediction); the dbsnp id (dbsnp_id); the severity of the variant (impact_severity); the presence or absence in the Catalogue Of Somatic Mutations In Cancer (COSMIC, http://cancer.sanger.ac.uk/cosmic); the presence or absence of the variant in the 1000 genomes project data (http://www.1000genomes.org/); the sample id (samples). See Supplementary Table 4

Supplementary Table 5: List of somatic loci. List of genomic loci corresponding to concordant or private mutations or wild type for each couple of the tree leaves reported in Figure 5. Sheet 1 "mutated loci in both": table reports loci mutated in both samples for each analyzed couple. Sheet 2 "only one mutated locus": table reports loci mutated in only one sample for each analyzed couple. Sheet 3 "none mutated locus": table reports wt loci for each analyzed couple. For each table: couple, samples of the couple; chrom, chromosome. The complete list of abbreviations used is reported in Supplementary Table 2. See Supplementary Table 5

Supplementary Table 6: Degree of similarity between ovary and matched synchronous lesions

Patient Code	Sample 1	Sample 2	concordant mutations	private mutations	wt
10152	omentum-synchr-metastasis	metachr-metastasis	0,75%	94,52%	4,73%
20144	douglas-synchr-metastasis	omentum-synchr-metastasis	0,46%	96,16%	3,38%
		omentum-metachr-metastasis	0,17%	97,11%	2,72%
	omentum-synchr-metastasis	omentum-metachr-metastasis	0,15%	97,07%	2,78%
20357	peritoneum-synchr-metastasis	douglas-metachr-metastasis	0,25%	95,70%	4,05%
20367	omentum-synchr-metastasis	omentum-metachr-metastasis	0,33%	95,50%	4,17%
20418	omentum-synchr-metastasis	peritoneum-metachr-metastasis	0,15%	95,43%	4,42%
20614	omentum-synchr-metastasis	colon-metachr-metastasis	0,12%	98,17%	1,70%
20654	omentum-synchr-metastasis	metachr-metastasis	0,31%	96,58%	3,11%
20663	omentum-synchr-metastasis	abdominal-wall-metachr-metastasis	0,17%	97,38%	2,45%
20681	synchr-metastasis	omentum-metachr-metastasis	0,21%	97,18%	2,62%
		right-hepatic-metachr-metastasis	0,46%	97,72%	1,83%
20683	omentum-synchr-metastasis	peritoneum-metachr-metastasis	0,71%	89,25%	10,05%
		colon-metachr-metastasis	0,44%	91,37%	8,20%
20738	diafram-synchr-metastasis	omentum-synchr-metastasis	0,42%	97,76%	1,83%
		douglas-metachr-metastasis	0,29%	98,11%	1,60%
		left ovary-metachr	0,44%	97,95%	1,62%
	omentum-synchr-metastasis	douglas-metachr-metastasis	0,35%	97,26%	2,39%
		left ovary-metachr	0,48%	97,07%	2,45%
20800	omentum-synchr-metastasis	peritoneum-metachr-metastasis	0,44%	93,65%	5,92%
20935	omentum-synchr-metastasis	omentum-metachr-metastasis	0,19%	97,30%	2,51%
20995	epiploic-appendix-synchr-metastasis	gastric-curvature-synchr-metastasis	0,23%	96,53%	3,24%
		ileum-nodules-synchr-metastasis	0,23%	95,58%	4,19%
		omentum-synchr-metastasis	0,19%	96,06%	3,76%
		stomach-metachr-metastasis	0,19%	95,31%	4,50%
	gastric-curvature-synchr-metastasis	ileum-nodules-synchr-metastasis	0,39%	96,53%	3,07%
		omentum-synchr-metastasis	0,23%	96,89%	2,89%
		stomach-metachr-metastasis	0,23%	96,14%	3,63%
	ileum-nodules-synchr-metastasis	omentum-synchr-metastasis	0,29%	95,99%	3,72%
		stomach-metachr-metastasis	0,25%	95,21%	4,55%
	omentum-synchr-metastasis	stomach-metachr-metastasis	0,10%	95,58%	4,32%
21019	omentum-synchr-metastasis	mesocolon-metachr-metastasis	0,33%	98,40%	1,27%
21110	abdominal-peritoneum-synchr-metastasis	prevesical-peritoneum-synchr-metastasis	0,37%	95,45%	4,17%
		mesoileum-metachr-metastasis	0,19%	96,01%	3,80%
	prevesical-peritoneum-synchr-metastasis	mesoileum-metachr-metastasis	0,21%	96,01%	3,78%
21184	omentum-synchr-metastasis	mesoileum-metachr-metastasis	0,08%	95,68%	4,23%
21296	synchr-metastasis	little-omentum-metachr-metastasis	0,12%	94,21%	5,67%

Table reports for each patient at primary surgery the degree of similarity (%) between tumor biopsies in the ovary and in matched tumor biopsies taken in different anatomical sites (synchronous lesions). Counts are based on allelic fractions as reported in https://github.com/lbeltrame/mnegri-ov198. Patient code, identification code of the patient. Sample 1 and Sample2, samples of the analyzed couple. Concordant mutations are mutations present in both sample of the couple, in the same genomic locus. Private mutations are mutations present in only one sample of the couple. Wt, wild type. The complete list of abbreviations used is reported in Supplementary Table 2.

Supplementary Table 7: Degree of similarity between ovary and its matched metachronous lesions

Patient Code	Sample 1	Sample 2	concordant mutations	private mutations	wt
10152	ovary	omentum-synchr-metastasis	0,87%	94,25%	4,88%
20144	ovary	douglas-synchr-metastasis	0,19%	97,30%	2,51%
		omentum-synchr-metastasis	0,15%	97,24%	2,62%
20357	right-ovary	peritoneum-synchr-metastasis	0,33%	97,07%	2,59%
20367	ovary	omentum-synchr-metastasis	0,33%	95,91%	3,76%
20418	ovary	omentum-synchr-metastasis	0,12%	98,11%	1,76%
20614	ovary	omentum-synchr-metastasis	0,12%	98,28%	1,60%
20654	ovary	omentum-synchr-metastasis	1,62%	98,38%	0,00%
20663	ovary	omentum-synchr-metastasis	0,15%	96,28%	3,57%
20681	ovary	synchr-metastasis	0,27%	97,70%	2,03%
20683	ovary	omentum-synchr-metastasis	3,59%	96,41%	0,00%
20724	left ovary	right-ovary	0,35%	97,97%	1,68%
20738	ovary	diafram-synchr-metastasis	0,35%	98,36%	1,29%
		omentum-synchr-metastasis	0,35%	97,45%	2,20%
20800	ovary	omentum-synchr-metastasis	0,48%	95,41%	4,11%
20935	ovary	omentum-synchr-metastasis	0,15%	96,76%	3,09%
20995	ovary	epiploic-appendix-synchr-metastasis	0,12%	96,51%	3,36%
		gastric-curvature-synchr-metastasis	0,12%	97,30%	2,57%
		ileum-nodules-synchr-metastasis	0,12%	96,35%	3,53%
		omentum-synchr-metastasis	0,08%	96,82%	3,09%
21019	ovary	omentum-synchr-metastasis	0,23%	98,07%	1,70%
21110	ovary	abdominal-peritoneum-synchr-metastasis	0,19%	96,66%	3,15%
		prevesical-peritoneum-synchr-metastasis	0,17%	96,62%	3,22%
21184	ovary	right-ovary	0,21%	94,04%	5,75%
		omentum-synchr-metastasis	0,21%	94,27%	5,52%
	right-ovary	omentum-synchr-metastasis	0,33%	94,96%	4,71%
21296	ovary	synchr-metastasis	0,23%	95,43%	4,34%

Table reports for each patient the degree of similarity (%) between tumor biopsies taken in the ovary and in matched tumor biopsies taken at relapse (metachronous lesions). Counts are based on the table of allelic fractions in https://github.com/lbeltrame/mnegri-ov198. Patient code, identification code of the patient. Sample 1 and Sample 2, samples of the analyzed couple. Concordant mutations are mutations present in both sample of the couple, in the same genomic locus. Private mutations are mutations present in only one sample of the couple. Wt, wild type. The complete list of abbreviations used is reported in Supplementary Table 2.

Supplementary Table 8: Degree of similarity between synchronous lesions and their matched metachronous lesions

Pat ìent Code	Sample 1	Sample 2	concordant mutat ìons	private mutat ìons	wt
10152	ovary	metachr-metastasis	0,98%	96,93%	2,10%
20144	ovary	omentum-metachr-metastasis	0,35%	98,69%	0,95%
20357	right-ovary	douglas-metachr-metastasis	0,66%	96,06%	3,28%
20367	ovary	omentum-metachr-metastasis	0,21%	95,93%	3,86%
20418	ovary	peritoneum-metachr-metastasis	0,19%	95,95%	3,86%
20614	ovary	colon-metachr-metastasis	0,21%	99,32%	0,48%
20654	ovary	metachr-metastasis	0,31%	96,58%	3,11%
20663	ovary	abdominal-wall-metachr-metastasis	0,12%	97,43%	2,45%
20681	ovary	omentum-metachr-metastasis	0,35%	97,45%	2,20%
		right-hepat ìc-metachr-metastasis	0,29%	97,68%	2,03%
20683	ovary	peritoneum-metachr-metastasis	0,71%	89,25%	10,05%
		colon-metachr-metastasis	0,44%	91,37%	8,20%
20724	lef tovary	mesovarium-metachr-metastasis	0,25%	97,55%	2,20%
		obturator-lymphnode-metachr-metastasis	0,27%	97,45%	2,28%
		pelvis-metachr-metastasis	0,27%	97,86%	1,87%
		peritoneum-metachr-metastasis	0,29%	97,99%	1,72%
		salpinx-metachr-metastasis	0,25%	98,09%	1,66%
	right-ovary	mesovarium-metachr-metastasis	0,56%	97,55%	1,89%
		obturator-lymphnode-metachr-metastasis	0,27%	97,14%	2,59%
		pelvis-metachr-metastasis	0,54%	97,82%	1,64%
		peritoneum-metachr-metastasis	0,39%	97,78%	1,83%
		salpinx-metachr-metastasis	0,48%	98,01%	1,52%
20738	ovary	douglas-metachr-metastasis	0,39%	97,97%	1,64%
		lef tovary-metachr	0,39%	97,65%	1,95%
20800	ovary	peritoneum-metachr-metastasis	0,54%	94,71%	4,75%
20935	ovary	omentum-metachr-metastasis	0,19%	97,03%	2,78%
20995	ovary	stomach-metachr-metastasis	0,21%	96,20%	3,59%
21019	ovary	mesocolon-metachr-metastasis	0,21%	98,44%	1,35%
21110	ovary	mesoileum-metachr-metastasis	0,17%	97,36%	2,47%
21184	ovary	mesoileum-metachr-metastasis	0,12%	94,94%	4,94%
	right-ovary	mesoileum-metachr-metastasis	0,08%	95,45%	4,46%
21296	ovary	lit ìte-omentum-metachr-metastasis	0,21%	93,81%	5,98%

Table reports for each patient the degree of similarity (%) between tumor biopsies taken at primary surgery in different anatomical sites (synchronous lesions) and at relapse (metachronous lesions). Counts are based on table of allelic fractions as reported in https://github.com/lbeltrame/mnegri-ov198. Patient code, identification code of the patient. Sample 1 and Sample 2, samples of the analyzed couple. Concordant mutations are mutations present in both sample of the couple, in the same genomic locus. Private mutations are mutations present in only one sample of the couple. Wt, wild type. The complete list of abbreviations used is reported in Supplementary Table 2.

SUPPLEMENTARY METHODS

1.1 Genomic DNA extraction and purification

Genomic DNA (gDNA) was extracted and automatically purified with an automatic extraction system (Maxwell® Rapid Sample Concentrator, Promega, Milan Italy). The concentration and the quality of gDNA was evaluated just before library preparation using Quant-iT™ PicoGreen® fluorescence assay (Thermo Fisher Scientific, Waltham, Massachusetts, USA).

2.1 Target re-sequencing libraries

TrueSeq Custom amplicons panel included 65 genes, arbitrary selected from the medical literature, on the basis of the presence of described mutations with an established role in response to targeted therapies and/or in current treatment paradigms. The complete list of genes with their chromosome coordinates, accordingly to USCS hg19 are as previously reported [4]. Design was performed by Design Studio algorithm following manufacturing instructions. In Supplementary Table 3, genes are organized into functional pathways based on Ingenuity Variant call Analysis software (Qiagen, Milan Italy). Color palette is used in downstream analysis to make visual analysis easier. Briefly, selected panel covers a total of 341748 bp, with a total of 2549 amplicons. 250 bp length amplicons were designed on the entire coding region, exon-intron boundaries (+/−10 bp); in addition, 2 Kb of the flanking sequence in the 5' UTR was added to the target region of selected genes [4]. Adaptor-ligated libraries were prepared from 250 ng of gDNA following protocol instructions (Illumina).

3.1 Sequencing data analysis

3.1.1 High level overview

Raw de-multiplexed reads from the MiSeq sequencer were aligned to the reference human genome (UCSC build hg19, the latest available when the targeted regions were designed) using the Burrows-Wheeler Aligner (BWA, version 0.7.12-r1039; [5]), running in paired-end mode. To ensure a good call quality and to reduce the number of false positives, samples underwent Base Quality Score Recalibration (BQSR), using the Genome Analysis Toolkit (GATK, version 3.3; [6–8]). After BQSR, sequences around regions with insertions and deletions (indels) were realigned locally using the Smith-Waterman algorithm. In this step, synchronous metastasis samples were recalibrated along with a blood counterpart acting as control,. Putative somatic variant calls were detected with two separate programs, MuTect (version 1.1.5; [9]) and VarScan 2 (version 2.3.6; [10]), pairing each sample with its matched blood.

Variant calls were annotated with biological information using the Variant Effect Predictor (VEP; version 81 with genome version hg19; [11]). Mutations were annotated with the 1000 Genomes project, dbSNP (version 138) and Catalogue of Somatic Mutations in Cancer (COSMIC), version 68. At the end of the analysis, variant calls from each sample pair were merged together using GATK, and subsequently filtered to exclude non-somatic calls, with an allelic fraction less than 1%, or with a read depth less than 200 fold (200×). A complete data set including all primary and synchronous lesions was built by merging the processed data with our previously published somatic variant calls [4].

The mutation data set was annotated with dbNSFP (version 3.2; [12]) and loaded into a database using the GEMINI software (version 0.17.0, [13]).

Variation and data are available on GitHub at https://github.com/lbeltrame/mnegri-ov190.

3.1.2 Detailed analysis methods

3.1.2.1 Analysis configuration

Synchronous metastases were analyzed using blood as normal control. A detailed sample sheet with individual sample configuration is available on GitHub. The analysis used the same gene panel as previously described [4]. In this case, FASTQ files from the two different panels were concatenated and all the regions from the two panels were assembled into a single BED file. Afterward, all samples were analyzed using the merged regions.

Marking of PCR duplicates was disabled. As additional configuration, we enabled clinical reporting for VEP in bcbio-nextgen, in order to provide amino acid and nucleotide changes in a format more easily understandable by clinicians.

The creation of per-sample GEMINI databases was disabled, as a more complete database was created after the analysis.

3.1.2.2 Analysis set-up and execution

FASTQ files and sample sheets were loaded on the two clusters part of the Cloud4CaRE project, a project between the Mario Negri Institute, the ACTO foundation and the computing departments of two major Italian banks to provide computational resources for NGS analysis. Cloud4CaRE is made up of two distinct computing platforms, a 214-CPU cluster (8 CPUs per node, 48 Gb RAM per node) or a 792-CPU cluster (24 CPUs per node, 48 Gb RAM per node), both running the Simple Linux Utility for Resource Management (SLURM) software for task scheduling.

Pre-computed genome indexes for BWA, along with genome sequences and associated files, were retrieved from the CloudBioLinux repository (http://cloudbiolinux.org) through bcbio-nextgen's upgrade command. The analysis pipeline was then run using bcbio-nextgen (version 0.9.2) on the two clusters (one for primary samples, and one for synchronous diseases).

Alignment coverage was measured with Chanjo (version 2.1.1) and BEDtools (version 2.24.0), wrapped in the calculate_coverages program.

3.1.2.3 Variant processing and annotation

VCF files produced by the variant callers were pre-filtered during the quality control steps, flagging non somatic variants with the REJECT flag. Per-sample allelic fraction measures (FA for MuTect and FREQ for VarScan 2) were converted into a standardized FREQ format field to increase compatibility with downstream tools.

After the completion of the pipeline, the final set of variant calls underwent further processing: calls from different samples were joined together using the GATK's CombineVariants walker, and afterwards all variants were joined together in a single file. Multi-allelic loci were decomposed into single alleles with the vt program (http://genome.sph.umich.edu/wiki/Vt) and subsequently annotated with the Variant Effect Predictor.

Lastly, variants from synchronous disease were combined with the data of matching, previously published primary tumors [4], using the CombineVariants walker from the GATK, giving locus priority, in case of identical loci, to the primary tumor data. Multi-allelic loci were then again decomposed with vt.

The final set of annotated variants was loaded into GEMINI for downstream analysis.

3.1.2.4 Phylogenetic tree reconstruction

Primary tumors and metachronous metastases from our previous work were re-analyzed with MuTect and VarScan as described in previous sections. Additionally, one sample from an unrelated ovarian tumor cell line (OVCAR-8) was analyzed using a pool of healthy controls as outgroup. Afterwards, fraction information was extracted from the VCF file and used to build a matrix where samples were represented in rows, and individual loci in columns. Missing data (NA) was given a fraction of 0.0. The data from the OVCAR-8 sample were also added to ensure a common point of origin for all samples. The matrix was generated with the PyVCF library (https://pyvcf.readthedocs.org/) coupled with the pandas data analysis package (version 0.17.1; [14]). Example code is available in the GitHub repository.

This matrix was then used to build distance measurements with the dist.gene function of the ape R package [15]. Individual sample groups were joined with a neighbor-joining tree estimation algorithm [16] as supplied by the nj function in the ape package. Trees were drawn with the plot.phylo function ("fan" tree type).

3.1.2.5 Data availability

The aligned sequences are available at the EBI European Nucleotide Archive (http://www.ebi.ac.uk/ena/data/view/; ID pending) and the configuration files, and all the associated programs (written in Python 2 and Python 3) are freely available on GitHub (https://github.com/lbeltrame/mnegri-ov198) under the GNU GPL or the BSD license. The complete joined mutation list is available as a Variant Call Format (VCF) file at https://github.com/lbeltrame/mnegri-ov198/tree/master/data.

3.1.2.6 Clustering

Hierarchical clustering was performed with the fastcluster package [16] coupled with the seaborn visualization library (https://stanford.edu/~mwaskom/software/seaborn/) to represent data as a heatmap. The chosen cluster metric was the Pearson's correlation coefficient, using complete linkage.

3.1.2.7 Pathway-based allelic fraction and statistical tests

From information on allelic fraction for each gene per sample, we calculated a normalized allelic fraction value (AF_{norm}) for each pathway per sample Given N as number of genes in the pathway and n as the number of mutated (with at least one non-synonymous mutation) genes inthe pathway, and AF_{mean} as the mean allelic fraction for all the genes in the pathway, $AF_{norm}$is expressed as:

$$AF_{norm} = (AF_{mean} * n) / N$$

These information were used to compare the main groups from the clustering analysis (see previous section), we evaluated AF_{norm} for each sample separately in both cluster groups we found, and significance of the difference was assessed by a t-test. p-values from the test were then corrected for multiple testing using the False Discovery Rate (FDR) method (Benjamini and Hochberg, 1995).

4. DNA ASSAY

4.1 BRCA1 DNA methylation assay

DNA methylation signatures of three different CpG island on BRCA1 promoter's region were analysed and quantified using pyrosequencing. Genomic DNA (500 ng) from each sample was bisulfite converted with the EpiTect Kit QIAGEN according to the manufacturer's recommendations. After purification, concentration of bisulfite DNA-treated was evaluated using the PicoGreen double-strand DNA assay kit (Invitrogen Life Technologies Quant-iT, Milan, Italy) and measured at fluorimeter (Infinite M200,TECAN, Milan, Italy), following manufacturer's instructions. Assay design was carried out using Qiagen Pyromark 2.0 software. Details of primer sequences and PCR are provided in Table 4.1.1.

PCR conditions were according to the Qiagen specifications with an annealing temperature of 61°C (experimentally determinate by a gradient PCR). After PCR, 5 µl of product was run on a 1% agarose gel to ascertain success of the reaction and successful reactions were taken forward to pyrosequencing. Pyrosequencing of methylated sites was performed using the PyroMark Q24 (QIAGEN) according to the manufacturer's protocol. Pyrosequencing runs were subjected to quality control using the Epitect® Plus DNA Bisulfite Kit QIAGEN to draw a standard curve calibration where interpolate data.

4.2 CCNE1 copy number variation assay

Copy number variation assays of CCNE1 have been performed using droplet digital pcr (ddPCR QX200™ Droplet Digital™ PCR System, Bio-Rad, Hercules, California, USA). Two reference gene probes (TERT and EIF2C1, 20X Dye Quencher 5′ HEX, Bio-Rad) have been used in order to normalized data obtained. PCR has been done in 20 µl with the following components (Bio-Rad, Hercules, California USA): 10 µl ddPCR™ Supermix for probes (no dUTPs) 2X, 0.8 µl PrimePCR ddPCR Copy Number Assay TERT 20X, 0.5 µl PrimePCR ddPCR Copy Number Assay EIF2C1 20X, 0.7 µl PrimePCR ddPCR Copy Number Assay CCNE1 (Dye Quencher 5′ FAM) 20X, 8 µl DNase/ RNase free- water and 1 µl of extracted DNA [5 ng/µl]. The PCR has been performed under specific thermal cycling conditions: 95° for 5 minutes, 95°C for 30 seconds and 59°C for 1 minute for 40 cycles, 98° for 10 minutes (ramping temperature 2°C/sec). Finally fluorescence has been detected and data analyzed by ddPCR QX™ Droplet Digital™ PCR System (Bio-Rad, Hercules, California, USA).

Table 4.1.1: Details of primer sequences and annealing temperature

BRCA1 promoter (chr17: 41277426-41277444)	
Forward PCR primer	GTTGTTTAGAGGTAGTTTTTTGGTTT
Reverse PCR primer	ACCTATCCCCCCTCCAAAAAATCTC
Sequence to analyze	RCRCTTTTCCRTTACCAC
Annealing Temperature	61°C
Sequencing Primer	CAATTTTAATTTATCTATAATTCCC

SUPPLEMENTARY REFERENCES

1. Beltrame L, Di Marino M, Fruscio R, Calura E, Chapman B, Clivio L, Sina F, Mele C, Iatropoulos P, Grassi T, Fotia V, Romualdi C, Martini P, et al. Profiling cancer gene mutations in longitudinal epithelial ovarian cancer biopsies by targeted next-generation sequencing: a retrospective study. Ann Oncol. 2015; 26:1363–71. https://doi.org/10.1093/annonc/mdv164. [PubMed]
2. Patch AM, Christie EL, Etemadmoghadam D, Garsed DW, George J, Fereday S, Nones K, Cowin P, Alsop K, Bailey PJ, Kassahn KS, Newell F, Quinn MC, et al, and Australian Ovarian Cancer Study Group. Whole-genome characterization of chemoresistant ovarian cancer. Nature. 2015; 521:489–94. https://doi.org/10.1038/nature14410. [PubMed]
3. Meier B, Cooke SL, Weiss J, Bailly AP, Alexandrov LB, Marshall J, Raine K, Maddison M, Anderson E, Stratton MR, Gartner A, Campbell PJ. C. elegans whole-genome sequencing reveals mutational signatures related to carcinogens and DNA repair deficiency. Genome Res. 2014; 24:1624–36. https://doi.org/10.1101/gr.175547.114. [PubMed]
4. Li H, Durbin R. Fast and accurate short read alignment with Burrows-Wheeler transform. Bioinformatics. 2009; 25:1754–60. https://doi.org/10.1093/bioinformatics/btp324. [PubMed]
5. McKenna A, Hanna M, Banks E, Sivachenko A, Cibulskis K, Kernytsky A, Garimella K, Altshuler D, Gabriel S, Daly M, DePristo MA. The Genome Analysis Toolkit: a MapReduce framework for analyzing next-generation DNA sequencing data. Genome Res. 2010; 20:1297–303. https://doi.org/10.1101/gr.107524.110. [PubMed]
6. DePristo MA, Banks E, Poplin R, Garimella KV, Maguire JR, Hartl C, Philippakis AA, del Angel G, Rivas MA, Hanna M, McKenna A, Fennell TJ, Kernytsky AM, et al. A framework for variation discovery and genotyping using next-generation DNA sequencing data. Nat Genet. 2011; 43:491–98. https://doi.org/10.1038/ng.806. [PubMed]
7. Van der Auwera GA, Carneiro MO, Hartl C, Poplin R, Del Angel G, Levy-Moonshine A, Jordan T, Shakir K, Roazen D, Thibault J, Banks E, Garimella KV, Altshuler D, et al. From FastQ data to high confidence variant calls: the Genome Analysis Toolkit best practices pipeline. Curr Protoc Bioinformatics. 2013; 43:11.10.1–11.10.33. https://doi.org/10.1002/0471250953.bi1110s43. [PubMed]
8. Cibulskis K, Lawrence MS, Carter SL, Sivachenko A, Jaffe D, Sougnez C, Gabriel S, Meyerson M, Lander ES, Getz G. Sensitive detection of somatic point mutations in impure and heterogeneous cancer samples. Nat Biotechnol. 2013; 31:213–19. https://doi.org/10.1038/nbt.2514. [PubMed]
9. Koboldt DC, Zhang Q, Larson DE, Shen D, McLellan MD, Lin L, Miller CA, Mardis ER, Ding L, Wilson RK. VarScan 2: somatic mutation and copy number alteration discovery in cancer by exome sequencing. Genome Res. 2012; 22:568–76. https://doi.org/10.1101/gr.129684.111. [PubMed]
10. McLaren W, Pritchard B, Rios D, Chen Y, Flicek P, Cunningham F. Deriving the consequences of genomic variants with the Ensembl API and SNP Effect Predictor. Bioinformatics. 2010; 26:2069–70. https://doi.org/10.1093/bioinformatics/btq330. [PubMed]
11. Liu X, Jian X, Boerwinkle E. dbNSFP v2.0: a database of human non-synonymous SNVs and their functional predictions and annotations. Hum Mutat. 2013; 34:E2393–402. https://doi.org/10.1002/humu.22376. [PubMed]
12. Paila U, Chapman BA, Kirchner R, Quinlan AR. GEMINI: integrative exploration of genetic variation and genome annotations. PLOS Comput Biol. 2013; 9:e1003153. https://doi.org/10.1371/journal.pcbi.1003153. [PubMed]
13. McKinney W. pandas: a Foundational Python Library for Data Analysis and Statistics. 2011; 10.
14. Paradis E, Claude J, Strimmer K. APE: Analyses of Phylogenetics and Evolution in R language. Bioinformatics. 2004; 20:289–90. https://doi.org/10.1093/bioinformatics/btg412. [PubMed]
15. Saitou N, Nei M. The neighbor-joining method: a new method for reconstructing phylogenetic trees. Mol Biol Evol. 1987; 4:406–25. https://doi.org/10.1093/oxfordjournals.molbev.a040454. [PubMed]
16. Müllner D. fastcluster: Fast Hierarchical, Agglomerative Clustering Routines for *R* and *Python*. J Stat Softw. 2013; 53. https://doi.org/10.18637/jss.v053.i09. Accessed July 13, 2021.
